# Long‐Term Efficacy and Safety of Glycerol Phenylbutyrate in Japanese Patients With Urea Cycle Disorders: Results From a Phase 3 Switch‐Over and 12‐Month Extension Study

**DOI:** 10.1002/jmd2.70082

**Published:** 2026-06-14

**Authors:** Yoichi Wada, Mahoko Furujo, Kenichi Kashimada, Takashi Hamazaki, Hiromi Nyuzuki, Keiko Ichimoto, Toshihiko Kakiuchi, Shirou Matsumoto, Yoriko Watanabe, Chiho Ono, Takako Shimizu, Hiroiku Furukawa, Kimitoshi Nakamura

**Affiliations:** ^1^ Department of Medical Science and Innovation SiRIUS Institute of Medical Research Sendai Japan; ^2^ Department of Pediatrics Tohoku University Hospital Sendai Japan; ^3^ Department of Pediatrics Okayama Medical Center Okayama Japan; ^4^ Division of Endocrinology and Metabolism National Center for Child Health and Development Tokyo Japan; ^5^ Department of Pediatrics Osaka Metropolitan University Osaka Japan; ^6^ Department of Pediatrics Niigata University Medical and Dental Hospital Niigata Japan; ^7^ Department of Metabolism Chiba Children's Hospital Chiba Japan; ^8^ Department of Pediatrics Saga University Saga Japan; ^9^ Department of Pediatrics Kumamoto University Kumamoto Japan; ^10^ Department of Pediatrics and Child Health Kurume University Kurume Japan; ^11^ OrphanPacific Inc. Tokyo Japan

**Keywords:** ammonia, glycerol phenylbutyrate, Japan, nitrogen scavenger, phenylbutyrate, rare disease, urea cycle disorder

## Abstract

Sodium phenylbutyrate (NaPBA) is used for nitrogen scavenging in urea cycle disorders (UCDs), but its volume, palatability, and sodium load affect adherence and ammonia control. Glycerol phenylbutyrate (GPB) offers an alternative option with demonstrated improvements in metabolic control and palatability. For a Japanese perspective, we undertook an open‐label, multicentre, prospective Phase 3 study of efficacy, pharmacokinetics (PK), and safety. In a switch‐over, 15 patients received NaPBA for 7 days, then GPB for 7 days. Primary endpoint: 24‐h blood ammonia AUC (AUC_NH3,0–24_). Secondary endpoints: blood ammonia and glutamine concentration; PK of scavenger metabolites in blood and urine; safety. Patients then received GPB for up to 12 months. For GPB and NaPBA, respectively, mean (SD) AUC_NH3,0–24_ was 627 (198) and 757 (307) μmol·h/L (ratio 0.849; 95% CI 0.723–0.997). Mean peak and mean blood ammonia were 37 and 26 μmol/L versus 52 and 32 μmol/L. Mean plasma AUC_0–24_ for phenylbutyrate (PBA), phenylacetate (PAA), and phenylacetylglutamine (PAGN) were 470, 1420, and 836 versus 425, 984, and 741 μg·h/mL. Mean PBA metabolite fluctuation was 297% versus 366%. One adverse event (hyperammonaemia) led to discontinuation in each treatment arm. In the extension (*n* = 14), mean (SD) blood ammonia at Month 12 was 21 (8) μmol/L. No new safety findings were observed. GPB demonstrated effective control of blood ammonia with a favourable safety profile. It offers a practical and clinically advantageous alternative to NaPBA, extending previous evidence to Japanese individuals with UCDs. Trial registration: jRCT2071220110.

## Synopsis

- In Japanese patients with urea cycle disorders, glycerol phenylbutyrate maintained ammonia control, was well tolerated, and exhibited prolonged coverage for ammonia scavenging, which could reduce the risk of subclinical and clinical hyperammonaemia.

## 1 Introduction

Urea cycle disorders (UCDs) are rare inherited metabolic conditions resulting from defects in the enzymes or transporters of the urea cycle, the primary pathway for removing excess nitrogen from the body [1]. When the cycle is disrupted, ammonia accumulates and can result in lethargy, vomiting, cerebral oedema, coma, and, in severe cases, death [2, 3].

Clinical severity varies depending on the degree of residual urea cycle function. Complete enzyme deficiencies typically present in the neonatal period with acute hyperammonaemic crises, while partial deficiencies may emerge later in life, often during episodes of catabolic stress [4, 5, 6, 7]. Outside of acute decompensations, patients may experience covert hyperammonaemia, linked to chronic neurocognitive impairment [8, 9, 10, 11].

Treatment strategies are designed to minimise the accumulation of ammonia and other nitrogenous waste. These include dietary protein restriction, essential amino acid supplements, and nitrogen‐scavenging agents. In some cases, liver transplantation is considered [12]. Although dietary protein restriction is a mainstay of treatment, many people with UCDs have an inherent aversion to protein or self‐limit their intake because of their fear of hyperammonaemia. This results in them failing to achieve the safe level of protein intake (SLPI) needed to support their growth, preserve lean muscle mass, and avoid catabolism [13, 14]. The capacity of the impaired nitrogen disposal pathway to dispose of nitrogen varies between and within individuals. Scavenger therapy is essential to buffer fluctuations in nitrogen load and help patients achieve their SLPI as clinically indicated [15].

Nitrogen‐scavenging drugs include sodium phenylbutyrate (NaPBA) tablets or granules for long‐term management of UCD. Phenylbutyrate (PBA) is metabolised to phenylacetate (PAA), which conjugates with glutamine to form phenylacetylglutamine (PAGN), excreted in the urine as an alternative route for nitrogen elimination [16]. PBA improves waste nitrogen excretion but also improves nitrogen homeostasis as a result of the generation of a reserve urea nitrogen synthetic capacity [17, 18]. However, NaPBA presents several limitations: poor palatability, high dosing volume, and high sodium content [19, 20]. These factors limit dose escalation, compromise adherence, and contribute to suboptimal ammonia control in clinical practice.

Glycerol phenylbutyrate (GPB; HPN‐100) is a triglyceride prodrug of PBA that was developed to address the limitations of NaPBA [21]. It is sodium‐free, more concentrated, and tasteless, and its slow intestinal hydrolysis provides prolonged PAA exposure and a more stable pharmacokinetic profile, meaning PAA is within the therapeutic range for longer [22, 23]. Clinical trials conducted in the US and Canadian populations found GPB offers at least equivalent, and in some cases superior, control of ammonia, with improved tolerability and quality of life [19, 20, 21, 24, 25].

In Japan, the estimated incidence of UCDs is 1 in 50 000 live births [26]. Persistent challenges include poor metabolic control, impaired growth, developmental delay, and reduced quality of life [3, 4, 5]. To date, no clinical trials have examined GPB in Japanese patients with UCDs. Variations in genetic background may influence pharmacokinetic responses and clinical outcomes.

We present findings from a Phase 3, open‐label study in Japanese paediatric and adult patients with UCDs. The study included a controlled switch‐over from NaPBA to GPB, followed by a 12‐month extension to assess the long‐term safety and efficacy of GPB in this population.

## 2 Methods

### 2.1 Study Design and Setting

This was a multicentre, phase 3, open‐label, one‐way switch‐over study with an extension period. It was initiated in April 2023 and was conducted at nine clinical sites across Japan. The analysis includes the switch‐over part and 12‐month data for the extension part, with data cut‐off in November 2024. The study was designed to evaluate the efficacy, pharmacokinetics, and safety of GPB compared with NaPBA in paediatric and adult patients with UCDs. Prior to enrolment, the study protocol, informed consent documents, and other relevant materials were reviewed and approved by the institutional review board at each participating site. Written informed consent was obtained from all participants or their guardians. The trial was registered in the Japan Registry of Clinical Trials under jRCT2071220110. The study was conducted in accordance with Good Clinical Practice guidelines, the ethical principles outlined in the Declaration of Helsinki, and applicable Japanese regulatory requirements. Written informed consent was obtained for all participants.

### 2.2 Participants

Eligible participants were male or female patients of any age with a confirmed diagnosis of UCD, based on genetic, enzymatic, or biochemical testing. For inclusion in the switch‐over phase, patients were required to have been on a stable dose of NaPBA for at least 1 week prior to Day 1, and to not have received sodium benzoate within 1 week prior to the start of study drug administration. Newly enrolled patients entering the extension phase were required to have not received NaPBA for at least 30 days before study drug administration. Exclusion criteria included a screening blood ammonia level of ≥ 100 μmol/L, signs and/or symptoms of hyperammonaemia within the previous 2 weeks, active infection or comorbidities likely to affect ammonia metabolism, clinical symptoms or laboratory test abnormalities that could increase the risk for participation in the study, intent to use drugs that could affect renal clearance or protein catabolism or other drugs known to increase blood ammonia levels, history of prolonged QT interval, history of hypersensitivity to PBA and/or phenylacetic acid, or history of liver transplantation.

### 2.3 Intervention

Both drugs were administered orally three times daily with or immediately after meals, although dosing frequency could be adjusted between 3 and 6 administrations per day in younger children to align with feeding schedules. The study consisted of two sequential treatment periods. In the 14‐day switch‐over part, participants received NaPBA at a dose and frequency determined on a subject‐by‐subject basis by the investigator at the screening visit for the first 7 days, followed by a direct switch to GPB at a molar‐equivalent dose at the same dosage and administration for Days 8–14. No changes to dose or frequency were permitted during this phase.

In the extension phase, participants who had completed the switch‐over part continued GPB at the same dose used during Days 8–14. Newly enrolled participants were GPB‐naïve, so they were initiated on GPB at 4.5 mL/m^2^/day then titrated within a range of 4.5–11.2 mL/m^2^/day, based on ammonia control, protein intake, and clinical symptoms. Dose adjustments were permitted at the investigator's discretion throughout the extension period.

### 2.4 Endpoints

The primary efficacy endpoint was the area under the curve (AUC) of blood ammonia concentration from 0 to 24 h (AUC_NH3,0–24_), assessed on Day 7 (end of NaPBA) and Day 14 (end of GPB). Secondary endpoints during the switch‐over part included the maximum (C_max_) and mean ammonia concentrations, number and percentage of patients with ammonia levels above the upper limit of normal (ULN), and glutamine concentrations. The ammonia level data were normalised to a standard laboratory reference range before conducting the analyses. This normalisation was done by applying a scale normalisation approach, using the formula s = x *(Us/Ux) where s is the normalised laboratory value, x is the original laboratory value, Ux is the upper limit of the normal reference range from the original laboratory, and Us is the upper limit of the normal reference range for the standard laboratory which is 35 μmol/L. Plasma concentrations of PBA, PAA, and PAGN were measured at predefined time points on Days 7 and 14. Key PK parameters included C_max_, C_min_, and AUC_0‐24_. Urinary excretion of PAGN (U‐PAGN) was also assessed, and the fraction of the administered PBA dose excreted as PAGN (Fe%) was calculated. In the extension phase, secondary endpoints included changes in mean blood ammonia and glutamine concentrations and the number and percentage of patients with ammonia levels above the ULN. Safety was evaluated throughout both phases, with assessments including treatment‐emergent adverse events (TEAEs), haematology and clinical chemistry, liver enzymes, vital signs, and electrocardiograms (ECGs).

### 2.5 Sample Size and Analysis Populations

The study planned to enrol approximately 10 evaluable patients for the switch‐over part and at least 15 patients for the extension phase, including at least five newly enrolled individuals. Seventeen patients were enrolled in the switch‐over and 15 entered the extension phase.

Three analysis populations were defined: the intention‐to‐treat (ITT) population, which was the primary analysis population for efficacy and PK parameters and included all patients who received at least one dose of both study drugs (NaPBA and GPB); the per‐protocol (PP) population, which consisted of subjects in the ITT population who had no major protocol deviations; and the safety population, which included all participants who received any amount of study drug (either NaPBA or GPB).

### 2.6 Statistical Analysis

Continuous variables were summarised using descriptive statistics (mean, standard deviation, median, minimum, and maximum); categorical variables were summarised as counts and percentages. Any confidence interval (CI) was calculated using appropriate statistical methods. Given the modest sample size, all inferential tests were considered exploratory.

The primary endpoint (AUC_NH3,0–24_) was log‐transformed and analysed using an analysis of variance (ANOVA) model, with treatment as a fixed effect and subject as a random effect. Differences in least square means, along with 90% and 95% confidence intervals, were calculated. Secondary efficacy and PK endpoints were summarised descriptively. Safety outcomes were summarised by system organ class and preferred term.

## 3 Results

### 3.1 Disposition and Baseline Characteristics

**FIGURE 1:**
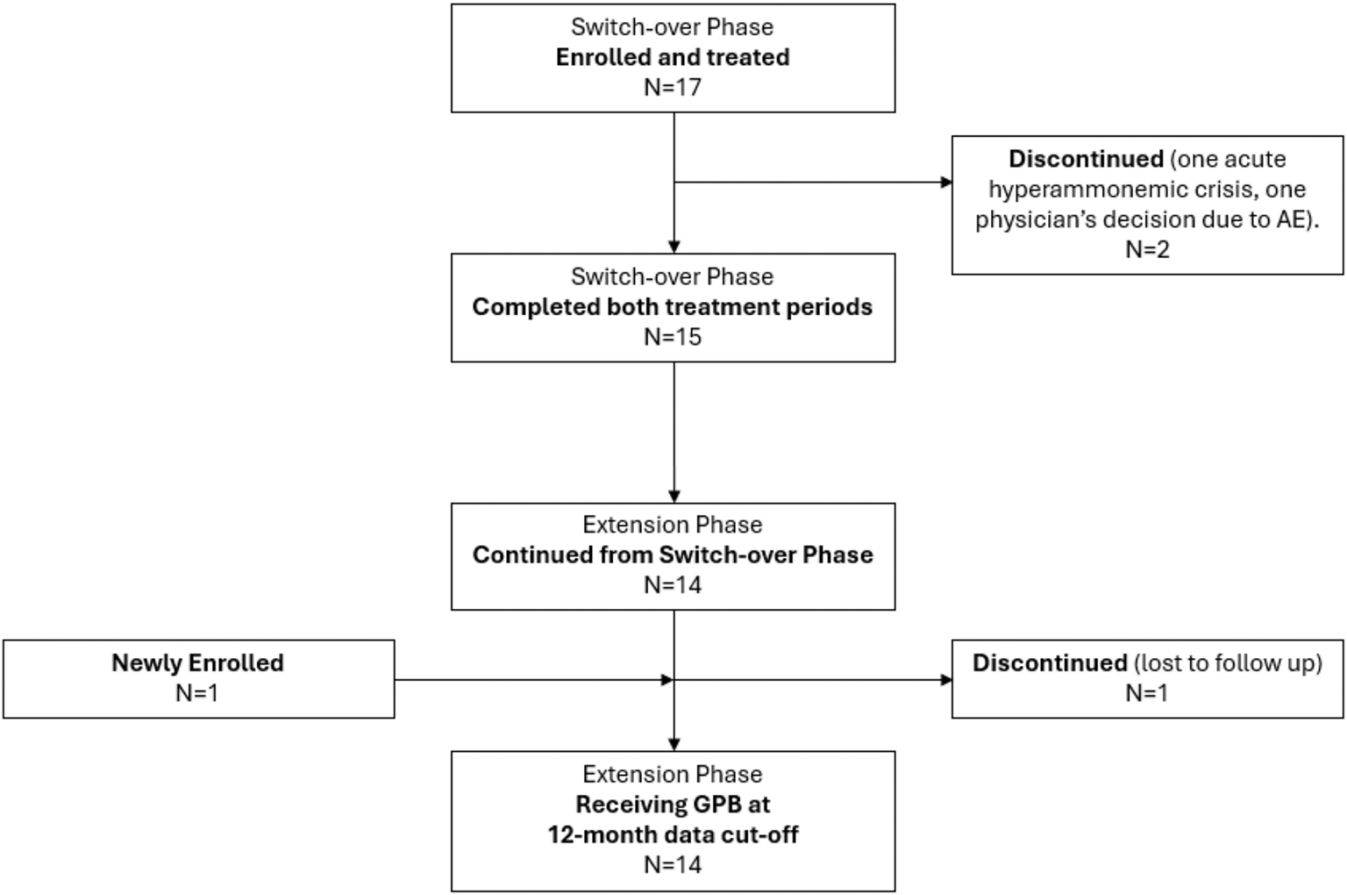
Study flow diagram.

Seventeen subjects were enrolled in the switch‐over phase of the study (Figure 1). Fifteen completed both treatment periods (NaPBA and GPB), while two withdrew prior to completion. The reason for discontinuation was an acute hyperammonaemic crisis for one patient and physician's decision due to an adverse event for the other. The ITT population comprised 16 subjects; one was excluded because they did not receive GPB. All 17 were included in the safety population.

Baseline characteristics are summarised in Table 1. Of the 17 subjects, 10 (59%) were under 18 years of age and 7 (41%) were adults, and 7 (41%) were male. Two (12%) were aged 2 to < 6 years, eight (47%) 6 to < 18 years, and seven (41%) adults aged 18 years or older. Diagnoses included ornithine transcarbamylase (OTC) deficiency in 9 patients (53%), argininosuccinate synthetase (ASS) deficiency in 6 (35%), and one case each (6%) of argininosuccinate lyase (ASL) and arginase (ARG) deficiency. The mean (SD) daily NaPBA dose at study entry was 10 (4.4) g, and the duration of prior NaPBA treatment was 94 (48) months. None of the subjects had experienced a recent hyperammonaemic crisis prior to enrolment.

**TABLE 1:** Demographics and diagnostic characteristics.

	Switch‐Over				
	All (*N* = 17)	0 ≤ < 2 (*N* = 0)	2 ≤ < 6 (*N* = 2)	6 ≤ < 18 (*N* = 8)	≥ 18 (*N* = 7)
Age group	*n* (%)	*n* (%)	*n* (%)	*n* (%)	*n* (%)
Sex					
Male	7 (41.2)	—	1 (50.0)	2 (25.0)	4 (57.1)
Female	10 (58.8)	—	1 (50.0)	6 (75.0)	3 (42.9)
History of hyperammonaemia					
Yes	6 (35.3)	—	0	5 (62.5)	1 (14.3)
No	11 (64.7)	—	2 (100.0)	3 (37.5)	6 (85.7)
UCD diagnosis					
ARG Deficiency	1 (5.9)	—	0	0	1 (14.3)
OTC Deficiency	9 (52.9)	—	1 (50.0)	3 (37.5)	5 (71.4)
ASS Deficiency	6 (35.3)	—	0	5 (62.5)	1 (14.3)
ASL Deficiency	1 (5.9)	—	1 (50.0)	0	0
UCD onset					
Neonatal (≤ 30 days)	7 (41.2)	—	1 (50.0)	5 (62.5)	1 (14.3)
Infantile (30 days≤ 2 years)	2 (11.8)	—	1 (50.0)	1 (12.5)	0
Childhood or Adult Onset (> 2 years)	8 (47.1)	—	0	2 (25.0)	6 (85.7)
NaPBA administration					
Yes	17 (100.0)	—	2 (100.0)	8 (100.0)	7 (100.0)
No	0	—	0	0	0
Duration of NaPBA treatment [months]					
*n*	17	0	2	8	7
Mean (S.D.)	93.8 (47.7)	‐ (−)	41.5 (9.2)	107.1 (53.2)	93.4 (40.0)
Median	109.0	—	41.5	105.0	112.0
[Min, Max]	[19, 213]	[−, −]	[35, 48]	[19, 213]	[32, 125]
Form of NaPBA treatment					
Powder	6 (35.3)	—	1 (50.0)	3 (37.5)	2 (28.6)
Tablet	11 (64.7)	—	1 (50.0)	5 (62.5)	5 (71.4)
Daily dose of NaPBA [g]					
*n*	17	0	2	8	7
Mean (S.D.)	10.3 (4.4)	‐ (−)	3.4 (0.6)	11.9 (4.1)	10.5 (3.5)
Median	10.5	—	3.39	12.5	10.5
[Min, Max]	[3.0, 18.0]	[−, −]	[3.0, 3.8]	[4.5, 18.0]	[6.0, 15.0]
Daily dose of NaPBA per body weight [g/kg]					
*n*	17	0	2	8	7
Mean (S.D.)	0.26 (0.11)	— (−)	0.29 (0.02)	0.30 (0.13)	0.21 (0.08)
Median	0.26	—	0.29	0.28	0.18
[Min, Max]	[0.09, 0.52]	[−, −]	[0.28, 0.30]	[0.16, 0.52]	[0.09, 0.33]
G‐tube					
Yes	2 (11.8)	—	1 (50.0)	1 (12.5)	0
No	15 (88.2)	—	1 (50.0)	7 (87.5)	7 (100.0)
Blood ammonia concentration at baseline [μmol/L]					
*n*	17	0	2	8	7
Mean (S.D.)	21.7 (10.13)	— (−)	20.8 (4.6)	19.96 (5.4)	23.94 (15.0)
Median	20.2	—	20.8	19.89	20.2
[Min, Max]	[10.08, 47.73]	[−, −]	[17.5, 24.1]	[10.1, 27.1]	[10.1, 47.7]
Hyperammonaemia at baseline					
Yes	0	—	0	0	0
No	17 (100.0)	—	2 (100.0)	8 (100.0)	7 (100.0)
Protein intake [g/day]					
*n*	14	0	1	7	6
Mean (S.D.)	45.7 (23.4)	— (−)	13.6 (−)	53.5 (29.5)	42.0 (8.8)
Median	45.0	—	13.6	48.0	45.0
[Min, Max]	[13.6, 113.8]	[−, −]	[13.6, 13.6]	[25.0, 113.8]	[30.0, 50.0]

Abbreviations: ARG = arginase; ASL = argininosuccinate lyase; ASS = argininosuccinate synthetase; BMI = body mass index; BSA = body surface area; DNA = deoxyribonucleic acid; G‐tube = gastrostomy tube; Max = maximum; Min = minimum; NaPBA = sodium phenylbutyrate; OTC = ornithine transcarbamylase; RBC = red blood cell; S.D. = standard deviation; UCD = urea cycle disorder. Maximum and mean blood ammonia concentration are based on the normalised ammonia results using the reference ULN of 35 μmol/L.

At the 12‐month data cut‐off, 14 subjects were receiving GPB, all of whom continued from the switch‐over period. One subject was newly enrolled in the extension part but was withdrawn because they were lost to follow‐up. Within the extension safety population (*n* = 15), 9 subjects (60%) were children and 6 (40%) were adults; 8 (53%) were male.

### 3.2 Dosing in the Switch‐Over Phase

During the switch‐over, the median total daily dose of GPB and NaPBA was 9.93 g/day and 10.25 g/day and ranged from 3.3 to 18.2 g/day and 3.0 to 18.0 g/day, respectively. Expressed by bodyweight, the median doses were identical: 236 mg/kg/day. The PBA content was very similar between the two regimens: a median of 9.18 g/day for GPB and 9.02 g/day for NaPBA.

### 3.3 Efficacy

**FIGURE 2:**
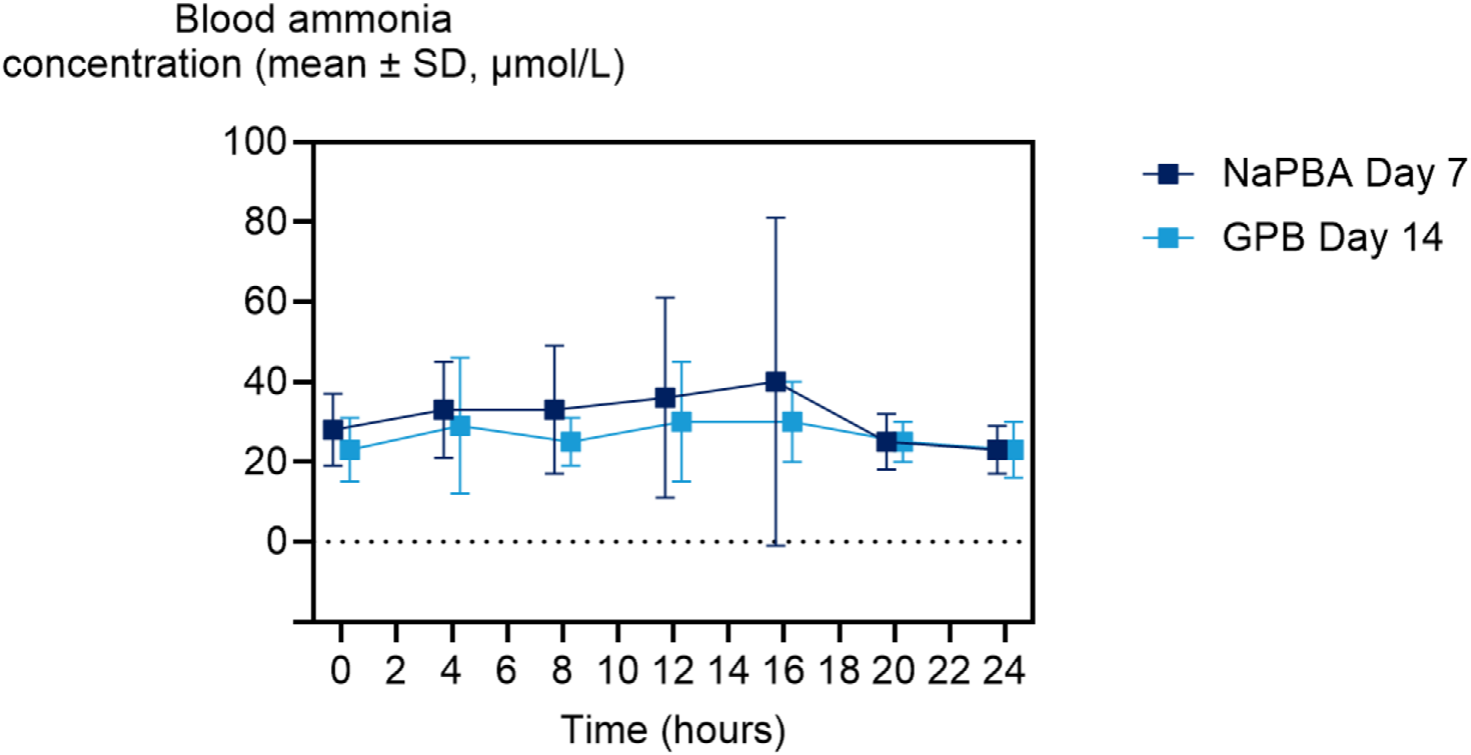
24‐h blood ammonia profiles (switch‐over phase) (all age groups). Measurement values are normalised ammonia results using the reference ULN of 35 μmol/L. SD = standard deviation; ULN = upper limit of normal.

In the switch‐over phase, the mean (SD) primary efficacy endpoint, AUC_NH3,0–24_, in the GPB and NaPBA groups, respectively, were 627 (198) and 757 (307) μmol·h/L (Table 2). Secondary efficacy outcomes reflected the primary findings (Table 3). The mean (SD) C_max_ of blood ammonia in the GPB and NaPBA groups was 37 (16) and 52 (38) μmol/L, and the ammonia concentration was 26 (8) and 32 (13) μmol/L, respectively. These treatment group patterns were similar in the paediatric subgroups aged 2 to < 6 and 6 to < 18 years, while values between treatments in adults were more similar to each other. The number of ammonia values exceeding the ULN of 35 μmol/L was relatively low during GPB treatment (Table S1). Between‐patient variability was also relatively low, as shown in the 24‐h blood ammonia time series plot (Figure 2). Mean (SD) blood concentrations of glutamine in the GPB and NaPBA groups were 604 (168) and 703 (186) μmol/L, respectively (Table S2).

**TABLE 2:** Primary ammonia outcome (switch‐over phase).

	Summary Statistics								ANOVA^a^				
	*n*	Mean (S.D.)	Min	Median	Max	CV (%)	Geometric Mean	Geometric CV (%)	Geometric LS Mean	Ratio of Geometric LS Mean (GPB/NaPBA)	90% CI	95% CI	p‐value
GPB	15	627.4 (197.9)	217	656.4	964	31.5	592.6	38.8	599.8	0.85	[0.74, 0.97]	[0.72, 1.00]	0.046
NaPBA	16	757.4 (306.8)	382	703.6	1447	40.5	706.6	39.2	706.6	—	—	—	—

*Note:* AUC is based on the normalised ammonia results using the reference ULN of 35 μmol/L. Lower and upper CI were exponentiated to express the results as original scale.

Abbreviations: ANOVA = analysis of variance; AUC = area under the concentration vs. time curve; AUC_NH3,0–24_ = area under the ammonia concentration versus time curve from hour 0 to 24 h; CI = confidence interval; CV = coefficient of variation; GPB = glycerol phenylbutyrate; LS mean = least squares mean; Max = maximum; Min = minimum; NaPBA = sodium phenylbutyrate; S.D. = standard deviation; ULN = upper limit of normal.

^a^ An ANOVA model for the natural log‐transformed AUC_NH3,0–24_ was constructed with factors for treatment as a fixed effect and subject as a random effect.

**TABLE 3:** Secondary ammonia outcomes (switch‐over phase).

	NaPBA (Day 7)	GPB (Day 14)
Age group: All		
Maximum blood ammonia concentration [μmol/L]		
*n*	16	15
Mean (S.D.)	52.3 (38.3)	37.3 (15.5)
Median	39.5	36.6
[Min, Max]	[22.8, 175.0]	[10.1, 71.6]
Mean blood ammonia concentration [μmol/L]		
*n*	16	15
Mean (S.D.)	32.0 (13.1)	26.3 (8.2)
Median	29.7	27.2
[Min, Max]	[16.2, 61.6]	[9.2, 40.1]
Age group: 2 ≤ < 6		
Maximum blood ammonia concentration [μmol/L]		
*n*	2	2
Mean (S.D.)	50.2 (8.8)	28.6 (2.2)
Median	50.2	28.6
[Min, Max]	[44.0, 56.4]	[27.1, 30.2]
Mean blood ammonia concentration [μmol/L]		
*n*	2	2
Mean (S.D.)	30.3 (0.94)	23.5 (2.09)
Median	30.3	23.5
[Min, Max]	[29.6, 30.9]	[22.1, 25.0]
Age group: 6 ≤ < 18		
Maximum blood ammonia concentration [μmol/L]		
*n*	8	7
Mean (S.D.)	62.4 (51.6)	36.9 (21.7)
Median	46.6	30.2
[Min, Max]	[22.8, 175.0]	[10.1, 71.6]
Mean blood ammonia concentration [μmol/L]		
*n*	8	7
Mean (S.D.)	33.7 (15.6)	24.6 (10.9)
Median	32.8	20.7
[Min, Max]	[16.2, 61.6]	[9.2, 40.1]
Age group: ≥ 18		
Maximum blood ammonia concentration [μmol/L]		
*n*	6	6
Mean (S.D.)	39.3 (16.6)	40.6 (8.0)
Median	35.0	42.2
[Min, Max]	[24.9, 71.6]	[27.1, 50.4]
Mean blood ammonia concentration [μmol/L]		
*n*	6	6
Mean (S.D.)	30.3 (12.8)	29.4 (5.2)
Median	25.2	29.8
[Min, Max]	[22.2, 55.9]	[20.7, 35.8]

*Note:* Maximum and mean blood ammonia concentration are based on the normalised ammonia results using the reference ULN of 35 μmol/L.

Abbreviations: GPB = glycerol phenylbutyrate; Max = maximum; Min = minimum; NaPBA = sodium phenylbutyrate; S.D. = standard deviation; ULN = upper limit of normal.

**FIGURE 3:**
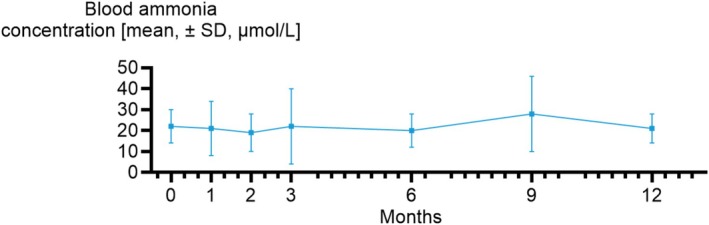
Blood ammonia over 12 months (extension phase). Measurement values are normalised ammonia results using the reference ULN of 35 μmol/L. SD = standard deviation; ULN = upper limit of normal.

In the extension phase, sustained control of blood ammonia concentrations was observed over the 12‐month period. These data are summarised in Figure 3, which shows monthly mean ammonia concentrations for the entire extension population. Mean values remained below the ULN across all visits, with the exception of a minor exceedance in patients aged 6 to < 18 years at Month 9 (Table S3).

### 3.4 Pharmacokinetics

Mean AUC_0‐24_ values for metabolites for the GPB and NaPBA groups were PBA (470 and 425 μg·h/mL), PAA (1420 and 984 μg·h/mL), and PAGN (836 and 741 μg·h/mL), respectively (Table 4). Fluctuation values [(C_max_‐C_min_)/C_av_] for PBA, PAA, and PAGN appeared relatively low with GPB treatment. U‐PAGN_0‐24_ was similar between GPB and NaPBA treatments (11 302 335 vs. 11 015 200 μg), as was the fraction of PBA excreted as PAGN (Fe_0‐24_: 73.5% vs. 75.0%) (Table S4). In the subgroup analysis by age, paediatric patients tended to show relatively higher exposure (AUC_0‐24_ and C_max_) to PAA and PAGN, whereas adults tended to have relatively higher exposure to PBA (Table S5).

**TABLE 4:** Plasma PK profiles (switch‐over phase).

	NaPBA (Day 7)					GPB (Day 14)				
	AUC_0‐24_[μg·hr/mL]	C_max_[μg/mL]	C_min_[μg/mL]	%Fluctuation	T_max_[hr]	AUC_0‐24_[μg·hr/mL]	C_max_[μg/mL]	C_min_[μg/mL]	%Fluctuation	T_max_[hr]
PBA										
*n*	16	16	16	16	16	15	15	15	15	15
Mean (S.D.)	425 (396)	62.1 (58.8)	0.499 (0.77)	365.96 (99.5)	11.36 (1.5)	470 (417)	59.7 (51.7)	0.702 (0.7)	296.53 (77.8)	9.82 (2.0)
PAA										
*n*	16	16	16	16	16	15	15	15	15	15
Mean (S.D.)	984 (969)	73.3 (61.9)	7.05 (17.9)	214.25 (71.7)	10.37 (3.4)	1420 (2060)	86.9 (92.4)	32.3 (74.4)	192.02 (92.4)	10.13 (3.7)
PAGN										
*n*	16	16	16	16	16	15	15	15	15	15
Mean (S.D.)	741 (389)	51.7 (22.9)	6.10 (7.2)	154.76 (39.1)	8.56 (2.8)	836 (518)	53.3 (23.9)	16.1 (22.6)	127.95 (55.1)	8.00 (3.2)

Abbreviations: GPB = glycerol phenylbutyrate; NaPBA = sodium phenylbutyrate; PAA = phenylacetate; PAGN = phenylacetylglutamine; PBA = phenylbutyrate; SD = standard deviation.

### 3.5 Safety

All 17 patients were evaluable for safety during the switch‐over part. TEAEs occurred in 6 of 16 patients (38%) receiving GPB and in 4 of 17 (24%) receiving NaPBA (Table 5). None of the TEAEs were considered to be related to the study drug. The majority of events were mild. One severe TEAE (hyperammonaemia) occurred during GPB treatment and led to treatment discontinuation. One mild and non‐serious event of hyperammonaemia was reported during NaPBA treatment and led to treatment discontinuation. Two patients experienced serious adverse events (feeling abnormal and hyperammonaemia) while on GPB; neither was judged to be drug‐related. No clinically significant abnormalities were observed in laboratory parameters, ECGs, or branched‐chain amino acid levels.

**TABLE 5:** Summary of adverse events (switch‐over and extension phases).

	NaPBA	GPB (switch‐over)	GPB (extension)
	*n* (%)	*n* (%)	*n* (%)
Age group: All	*N* = 17	*N* = 16	*N* = 15
TEAEs	4 (23.5)	6 (37.5)	14 (93.3)
Serious TEAEs	0	2 (12.5)	2 (13.3)
TEAEs related to study drug	0	0	2 (13.3)
TEAEs leading to death	0	0	0
TEAEs leading to discontinuation of study drug	1 (5.9)	1 (6.3)	0
Age group: 2 ≤ < 6	(*N* = 2)	(*N* = 2)	*N* = 2
TEAEs	0	1 (50.0)	2 (100.0)
Serious TEAEs	0	0	1 (50.0)
TEAEs related to study drug	0	0	0
TEAEs leading to death	0	0	0
TEAEs leading to discontinuation of study drug	0	0	0
Age group: 6 ≤ < 18	(*N* = 8)	(*N* = 8)	*N* = 7
TEAEs	3 (37.5)	3 (37.5)	7 (100.0)
Serious TEAEs	0	1 (12.5)	1 (14.3)
TEAEs related to study drug	0	0	1 (14.3)
TEAEs leading to death	0	0	0
TEAEs leading to discontinuation of study drug	0	1 (12.5)	0
Age group: ≥ 18	(*N* = 7)	(*N* = 6)	*N* = 6
TEAEs	1 (14.3)	2 (33.3)	5 (83.3)
Serious TEAEs	0	1 (16.7)	0
TEAEs related to study drug	0	0	1 (16.7)
TEAEs leading to death	0	0	0
TEAEs leading to discontinuation of study drug	1 (14.3)	0	0

Abbreviations: GPB = glycerol phenylbutyrate; TEAE = treatment‐emergent adverse event.

In the extension period, 14 of 15 patients (93%) reported TEAEs, most of which were mild or moderate in intensity (Table 5). Two events (nausea and QT prolongation) were considered treatment‐related. One severe event (gastroenteritis norovirus) and two episodes of hyperammonaemia occurred, all of which were deemed unrelated to GPB. No patients discontinued the study due to adverse events, and no deaths were reported. Clinical laboratory values, ECGs, and amino acid profiles remained stable throughout long‐term follow‐up.

## 4 Discussion

This study provides novel prospective evidence confirming the effectiveness of GPB in Japanese patients with UCDs. Treatment with GPB was associated with 24‐h ammonia AUC values consistent with tight ammonia control. The findings are scientifically robust and align with the established mechanism of GPB. The sustained 24‐h ammonia control observed here aligns with results from prior international trials in both children and adults, supporting GPB's role in reducing the risks of clinical hyperammonaemia and subclinical ammonia excursions [22]. In a larger long‐term study including 45 paediatric and 43 adult patients, mean ammonia levels remained stable and below the adult ULN (< 35 μmol/L) over 24 months of GPB treatment, while glutamine levels decreased over time in the overall population [27].

Our pharmacokinetic findings also provide evidence for GPB's effectiveness. The AUC_0‐24_ values for PBA, PAA, and PAGN were relatively high, while intra‐dosing fluctuations were low, indicating stable systemic exposure [27, 28]. This profile is consistent with tight ammonia control and reflects GPB's mechanism of gradually releasing PAA, which enables steady PAGN production—the principal route of nitrogen excretion. Prior phase 2 studies in adults demonstrated approximately 30% lower ammonia exposure with GPB versus NaPBA and a strong correlation between U‐PAGN and blood ammonia [28]. The present study confirms this relationship in Japanese patients, showing that consistent PAGN generation with GPB translates into stable and effective ammonia control throughout the dosing interval. These findings close an important evidence gap by confirming in a prospective setting that GPB provides effective ammonia control and consistent pharmacokinetics in Japanese patients with UCDs.

To achieve stable ammonia levels over the long term, it is necessary to have a scavenger that patients can adhere to and which can be prescribed at an adequate dose. Dose escalation can be a problem with the sodium‐based scavengers NaPBA and sodium benzoate (NaBz) because of their high sodium content and large dosing volumes. In addition, both scavengers have poor palatability; for example, NaPBA is poorly tolerated because of its bitter taste. These formulation shortcomings are known to affect adherence and have been reported as contributing to hyperammonaemic episodes in UCDs [29]. GPB, by contrast, is a liquid formulation with virtually no taste or odour, directly addressing these issues. As an oral liquid with low volume, GPB is generally easier to administer, particularly in paediatric patients or those with feeding difficulties. Its palatability may reduce the risk of dose refusal or incomplete administration, supporting consistent intake. GPB also eliminates the sodium burden linked to NaPBA; this is an important factor given the cardiovascular and renal risks associated with high sodium intake, especially in patients requiring lifelong therapy. Together, these formulation and dosing characteristics can reduce caregiver burden, improve overall treatment tolerability, and support the use of greater scavenger activity via higher doses where appropriate to achieve effective ammonia control.

A primary clinical objective in UCD management is ensuring patients achieve the SLPI to prevent catabolism and support growth. NaPBA's poor palatability, high volume, and sodium burden can hinder patient adherence and attainment of target dietary protein intakes. Although our study did not directly quantify changes in food intake, the stable metabolic control observed—combined with patients following SLPI‐aligned diets—suggests that GPB could allow for greater dietary protein intake in selected patients while maintaining satisfactory biochemical control. This aligns with data showing that patients on GPB can maintain normal protein and calorie intake with stable ammonia and urinary nitrogen waste excretion [19, 20, 25, 28, 30]. Such pharmacological stability may help support patients, their families and the broader healthcare team, including dietitians, in achieving at least the SLPI.

The safety profile of GPB in the study was consistent with expectations; adverse events were mostly mild, with one unrelated hyperammonaemic event during switch‐over, and no discontinuations during the 12‐month extension. This is in keeping with the broader GPB safety experience, which includes favourable tolerability, relatively few hyperammonaemic crises, and no new safety signals in long‐term open‐label studies [21, 22, 23, 27, 31, 32]. Compared with earlier generation nitrogen scavengers, GPB's improved palatability, lower dose volume, and sodium‐free formulation have also been associated with enhanced treatment satisfaction and adherence by healthcare professionals and patients/carers [20, 33, 34].

UCDs are rare in Japan (approximately 1 in 50 000 births), and NaPBA remains the most widely used nitrogen scavenger. However, the data presented here support a transition to GPB, showing that it provides tight ammonia control without sacrificing safety. The structured switch‐over in this study demonstrated that patients on stable NaPBA therapy can be transitioned directly to GPB using a molar‐equivalent dose, with no need for a titration period—consistent with previous international studies [19, 24, 25]. These findings align with global evidence and provide a foundation for integrating GPB into Japanese clinical guidelines. One aspect for particular consideration locally is that GPB contains glycerol (~15% w/w) and citrin deficiency—relatively common in Asia—is contraindicated for glycerol use due to potential worsening of hepatic NADH/NAD^+^ imbalance.

Limitations of the current study include its open‐label design and modest sample size, determined by the rarity of UCDs. While these constraints limit statistical power, the directionality and consistency of the results across key efficacy and safety outcomes are compelling. Importantly, future research should incorporate patient‐reported outcomes, quality‐of‐life assessments, and health economic data to capture the broader impact of GPB, including adherence, caregiver burden, and healthcare resource utilisation. Moreover, long‐term neurocognitive outcomes, especially following early‐life GPB exposure, warrant further study, given evidence of improved executive function in paediatric patients [22].

## 5 Conclusion

In Japanese patients with UCDs, GPB provided effective and sustained ammonia control and a favourable safety profile. These findings, consistent with international data, support GPB's inclusion in standard UCD management practice in Japan. Compared with NaPBA, GPB offers practical advantages—improved palatability, lower volume, and the absence of sodium—that will likely support adherence and long‐term treatment continuity. Given the need for lifelong metabolic control in UCDs, GPB's formulation and pharmacological profile present a valuable option for supporting chronic disease management in both new and existing patients.

## Author Contributions

Y.W., M.F., K.K., T.H., H.N., K.I., T.K., S.M., Y.Wa., and K.N. were involved in patient recruitment and data collection. C.O., T.S., and H.F. contributed to study design, data analysis, and manuscript preparation. All authors critically revised the manuscript and reviewed and approved the final manuscript.

## Funding

This work was supported by OrphanPacific Inc. Medical writing support was provided by Dr Paul Riley, PhD of Nexcea, Manchester, UK, funded by OrphanPacific Inc.

## Ethics Statement

The study was approved by the institutional review board at each participating site.

## Consent

Written informed consent was obtained from all participants or their legal guardians prior to enrolment in the study.

## Conflicts of Interest

C.O., T.S., and H.F. are employees of OrphanPacific Inc., the sponsor of the study. The remaining authors declare no conflicts of interest.

## Supporting information


**Table S1:** Ammonia excursions (switch‐over phase).

**Table S2:** Glutamine levels (switch‐over phase).

**Table S3:** Mean ammonia values during the extension phase.

**Table S4:** Summary of PK parameters of PBA, PAA, and PAGN (switch‐over, intent‐to‐treat population).

**Table S5:** Summary of PK parameters of PAA, PBA, and PAGN by age (switch‐over, intent‐to‐treat population).
**Table S5a.** Age group: 0 ≤ < 2.
**Table S5b.** Age group: 2 ≤ < 6.
**Table S5c.** Age group: 6 ≤ < 18.
**Table S5d.** Age group: ≥ 18.

**Data S1:** Plain language summary in Japanese.

**Data S2:** Plain language summary in English.

## Data Availability

Data supporting the findings of this study are available from the corresponding author upon reasonable request. Individual participant data will be de‐identified and shared in accordance with applicable data protection laws and institutional policies.

## References

[1] S. W. Brusilow and N. E. Maestri , “Urea Cycle Disorders: Diagnosis, Pathophysiology, and Therapy,” Advances in Pediatrics 43 (1996): 127–170. 8794176

[2] O. Braissant , V. A. McLin , and C. Cudalbu , “Ammonia Toxicity to the Brain,” Journal of Inherited Metabolic Disease 36, no. 4 (July 2013): 595–612. 23109059 10.1007/s10545-012-9546-2

[3] T. Uchino , F. Endo , and I. Matsuda , “Neurodevelopmental Outcome of Long‐Term Therapy of Urea Cycle Disorders in Japan,” Journal of Inherited Metabolic Disease 21, no. S1 (June 1998): 151–159. 9686352 10.1023/a:1005374027693

[4] J. Kido , S. Matsumoto , J. Häberle , et al., “Long‐Term Outcome of Urea Cycle Disorders: Report From a Nationwide Study in Japan,” Journal of Inherited Metabolic Disease 44, no. 4 (July 2021): 826–837. 33840128 10.1002/jimd.12384

[5] J. Kido , S. Matsumoto , T. Ito , et al., “Physical, Cognitive, and Social Status of Patients With Urea Cycle Disorders in Japan,” Molecular Genetics and Metabolism Reports 27 (June 2021): 100724. 33614409 10.1016/j.ymgmr.2021.100724 PMC7876628

[6] K. Nakamura , J. Kido , S. Matsumoto , H. Mitsubuchi , and F. Endo , “Clinical Manifestations and Growth of Patients With Urea Cycle Disorders in Japan,” Journal of Human Genetics 61, no. 7 (July 2016): 613–616. 26935171 10.1038/jhg.2016.17

[7] K. M. Stepien , T. Geberhiwot , C. J. Hendriksz , and E. P. Treacy , “Challenges in Diagnosing and Managing Adult Patients With Urea Cycle Disorders,” Journal of Inherited Metabolic Disease 42, no. 6 (November 2019): 1136–1146. 30932189 10.1002/jimd.12096

[8] M. L. Batshaw , Y. Roan , A. L. Jung , L. A. Rosenberg , and S. W. Brusilow , “Cerebral Dysfunction in Asymptomatic Carriers of Ornithine Transcarbamylase Deficiency,” New England Journal of Medicine 302, no. 9 (February 1980): 482–485. 7351973 10.1056/NEJM198002283020902

[9] K. Gyato , J. Wray , Z. J. Huang , M. Yudkoff , and M. L. Batshaw , “Metabolic and Neuropsychological Phenotype in Women Heterozygous for Ornithine Transcarbamylase Deficiency,” Annals of Neurology 55, no. 1 (January 2004): 80–86. 14705115 10.1002/ana.10794

[10] O. Braissant , “Current Concepts in the Pathogenesis of Urea Cycle Disorders,” Molecular Genetics and Metabolism 100 (January 2010): S3–S12. 20227314 10.1016/j.ymgme.2010.02.010

[11] C. Sprouse , J. King , G. Helman , et al., “Investigating Neurological Deficits in Carriers and Affected Patients With Ornithine Transcarbamylase Deficiency,” Molecular Genetics and Metabolism 113, no. 1–2 (September 2014): 136–141. 24881970 10.1016/j.ymgme.2014.05.007 PMC4458385

[12] J. Häberle , A. Burlina , A. Chakrapani , et al., “Suggested Guidelines for the Diagnosis and Management of Urea Cycle Disorders: First Revision,” Journal of Inherited Metabolic Disease 42, no. 6 (November 2019): 1192–1230. 30982989 10.1002/jimd.12100

[13] Joint WHO/FAO/UNU Expert Consultation , “Protein and Amino Acid Requirements in Human Nutrition,” World Health Organization Technical Report Series, no. 935 (2007): 1–265, back cover. 18330140

[14] S. Adam , M. F. Almeida , M. Assoun , et al., “Dietary Management of Urea Cycle Disorders: European Practice,” Molecular Genetics and Metabolism 110, no. 4 (December 2013): 439–445. 24113687 10.1016/j.ymgme.2013.09.003

[15] F. Feillet and J. V. Leonard , “Alternative Pathway Therapy for Urea Cycle Disorders,” Journal of Inherited Metabolic Disease 21, no. S1 (June 1998): 101–111. 9686348 10.1023/a:1005365825875

[16] F. Scaglia , “New Insights in Nutritional Management and Amino Acid Supplementation in Urea Cycle Disorders,” Molecular Genetics and Metabolism 100 (January 2010): S72–S76. 20299258 10.1016/j.ymgme.2010.02.019 PMC4831209

[17] S. W. Brusilow and J. Finkelstien , “Restoration of Nitrogen Homeostasis in a Man With Ornithine Transcarbamylase Deficiency,” Metabolism 42, no. 10 (October 1993): 1336–1339. 8412748 10.1016/0026-0495(93)90135-b

[18] B. Lee , H. Yu , F. Jahoor , W. O'Brien , A. L. Beaudet , and P. Reeds , “ *In Vivo* Urea Cycle Flux Distinguishes and Correlates With Phenotypic Severity in Disorders of the Urea Cycle,” National Academy of Sciences of the United States of America 97, no. 14 (July 2000): 8021–8026. 10.1073/pnas.140082197 PMC16663 10869432

[19] M. Yeo , P. Rehsi , M. Dorman , et al., “Clinical Experience With Glycerol Phenylbutyrate in 20 Patients With Urea Cycle Disorders at a UK Paediatric Centre,” JIMD Reports 64, no. 5 (September 2023): 317–326. 37701329 10.1002/jmd2.12386 PMC10494499

[20] G. Yeowell , D. S. Burns , and F. Fatoye , “The Burden of Pharmacological Treatment on Health‐Related Quality of Life in People With a Urea Cycle Disorder: A Qualitative Study,” Journal of Patient‐Reported Outcomes 5, no. 1 (December 2021): 110. 34694515 10.1186/s41687-021-00387-x PMC8546029

[21] N. Longo and R. J. Holt , “Glycerol Phenylbutyrate for the Maintenance Treatment of Patients With Deficiencies in Enzymes of the Urea Cycle,” Expert Opinion on Orphan Drugs 5, no. 12 (December 2017): 999–1010.

[22] G. A. Diaz , L. S. Krivitzky , M. Mokhtarani , et al., “Ammonia Control and Neurocognitive Outcome Among Urea Cycle Disorder Patients Treated With Glycerol Phenylbutyrate,” Hepatology 57, no. 6 (June 2013): 2171–2179. 22961727 10.1002/hep.26058 PMC3557606

[23] S. A. Berry , J. Vockley , A. A. Vinks , et al., “Pharmacokinetics of Glycerol Phenylbutyrate in Pediatric Patients 2 Months to 2 Years of Age With Urea Cycle Disorders,” Molecular Genetics and Metabolism 125, no. 3 (November 2018): 251–257. 30217721 10.1016/j.ymgme.2018.09.001

[24] M. Yeo , P. Rehsi , M. Dorman , et al., “Direct Replacement of Oral Sodium Benzoate With Glycerol Phenylbutyrate in Children With Urea Cycle Disorders,” JIMD Reports 63, no. 2 (March 2022): 137–145. 35281661 10.1002/jmd2.12274 PMC8898712

[25] E. Martín‐Hernández , P. Quijada‐Fraile , P. Correcher , et al., “Switching to Glycerol Phenylbutyrate in 48 Patients With Urea Cycle Disorders: Clinical Experience in Spain,” Journal of Clinical Medicine 11, no. 17 (August 2022): 5045. 36078975 10.3390/jcm11175045 PMC9457033

[26] N. Nagata , I. Matsuda , and K. Oyanagi , “Estimated Frequency of Urea Cycle Enzymopathies in Japan,” American Journal of Medical Genetics 39, no. 2 (May 1991): 228–229. 2063931 10.1002/ajmg.1320390226

[27] G. A. Diaz , A. Schulze , N. Longo , et al., “Long‐Term Safety and Efficacy of Glycerol Phenylbutyrate for the Management of Urea Cycle Disorder Patients,” Molecular Genetics and Metabolism 127, no. 4 (August 2019): 336–345. 31326288 10.1016/j.ymgme.2019.07.004

[28] B. Lee , W. Rhead , G. A. Diaz , et al., “Phase 2 Comparison of a Novel Ammonia Scavenging Agent With Sodium Phenylbutyrate in Patients With Urea Cycle Disorders: Safety, Pharmacokinetics and Ammonia Control,” Molecular Genetics and Metabolism 100, no. 3 (July 2010): 221–228. 20382058 10.1016/j.ymgme.2010.03.014 PMC2905228

[29] O. A. Shchelochkov , K. Dickinson , B. F. Scharschmidt , B. Lee , M. Marino , and C. Le Mons , “Barriers to Drug Adherence in the Treatment of Urea Cycle Disorders: Assessment of Patient, Caregiver and Provider Perspectives,” Molecular Genetics and Metabolism Reports 8 (September 2016): 43–47. 27493880 10.1016/j.ymgmr.2016.07.003 PMC4963256

[30] J. P. R. Monteleone , M. Mokhtarani , G. A. Diaz , et al., “Population Pharmacokinetic Modeling and Dosing Simulations of Nitrogen‐Scavenging Compounds: Disposition of Glycerol Phenylbutyrate and Sodium Phenylbutyrate in Adult and Pediatric Patients With Urea Cycle Disorders,” Journal of Clinical Pharmacology 53, no. 7 (July 2013): 699–710. 23775211 10.1002/jcph.92 PMC3923458

[31] S. A. Berry , U. Lichter‐Konecki , G. A. Diaz , et al., “Glycerol Phenylbutyrate Treatment in Children With Urea Cycle Disorders: Pooled Analysis of Short and Long‐Term Ammonia Control and Outcomes,” Molecular Genetics and Metabolism 112, no. 1 (May 2014): 17–24. 24630270 10.1016/j.ymgme.2014.02.007 PMC4382922

[32] S. A. Berry , N. Longo , G. A. Diaz , et al., “Safety and Efficacy of Glycerol Phenylbutyrate for Management of Urea Cycle Disorders in Patients Aged 2 Months to 2 Years,” Molecular Genetics and Metabolism 122, no. 3 (November 2017): 46–53. 28916119 10.1016/j.ymgme.2017.09.002

[33] K. M. Stepien , M. McSweeney , A. Ochoa‐Ferraro , R. Vara , P. Riley , and M. Smith , “Perspectives on Long‐Term Medical Management of Urea Cycle Disorders: Insights From a Survey of UK Healthcare Professionals,” Orphanet Journal of Rare Diseases 20, no. 1 (March 2025): 135. 40102865 10.1186/s13023-025-03647-x PMC11921535

[34] G. M. Enns , M. H. Porter , M. Francis‐Sedlak , A. Burdett , and J. Vockley , “Perspectives on Urea Cycle Disorder Management: Results of a Clinician Survey,” Molecular Genetics and Metabolism 128, no. 1–2 (September 2019): 102–108. 31377149 10.1016/j.ymgme.2019.07.009

