# Supplementary material for: Long‐Term Efficacy and Safety of Glycerol Phenylbutyrate in Japanese Patients With Urea Cycle Disorders: Results From a Phase 3 Switch‐Over and 12‐Month Extension Study
Source: JIMD Rep. 2026 Jun 14;67(4):e70082. doi: 10.1002/jmd2.70082 (PMC13265243; doi:10.1002/jmd2.70082)
Supplement: Supplementary file 1 — Table S1: Ammonia excursions (switch‐over phase). [file JMD2-67-e70082-s001.docx]

**Supplementary Table 1. Ammonia Excursions (Switch-Over Phase)**

|  | NaPBA (Day 7) | | GPB (Day 14) | |
| --- | --- | --- | --- | --- |
|  | N | n (%) | N | n (%) |
| Age group: All |  |  |  |  |
| Number and percentage of subjects with blood ammonia levels > ULN |  |  |  |  |
| 0 hr (pre-dose) | 15 | 3 (20.0) | 15 | 1 (6.7) |
| 4 hr | 14 | 4 (28.6) | 13 | 2 (15.4) |
| 8 hr | 16 | 6 (37.5) | 15 | 1 (6.7) |
| 12 hr | 16 | 6 (37.5) | 15 | 5 (33.3) |
| 16 hr | 14 | 5 (35.7) | 13 | 5 (38.5) |
| 20 hr | 14 | 1 (7.1) | 13 | 0 (0.0) |
| 24 hr | 16 | 1 (6.3) | 15 | 1 (6.7) |
| Any time point | 16 | 10 (62.5) | 15 | 8 (53.3) |
|  |  |  |  |  |
| Age group: 2<= <6 |  |  |  |  |
| Number and percentage of subjects with blood ammonia levels > ULN |  |  |  |  |
| 0 hr (pre-dose) | 2 | 0 (0.0) | 2 | 0 (0.0) |
| 8 hr | 2 | 2 (100.0) | 2 | 0 (0.0) |
| 12 hr | 2 | 0 (0.0) | 2 | 0 (0.0) |
| 24 hr | 2 | 0 (0.0) | 2 | 0 (0.0) |
| Any time point | 2 | 2 (100.0) | 2 | 0 (0.0) |
|  |  |  |  |  |
| Age group: 6<= <18 |  |  |  |  |
| Number and percentage of subjects with blood ammonia levels > ULN |  |  |  |  |
| 0 hr (pre-dose) | 7 | 2 (28.6) | 7 | 0 (0.0) |
| 4 hr | 8 | 3 (37.5) | 7 | 2 (28.6) |
| 8 hr | 8 | 3 (37.5) | 7 | 0 (0.0) |
| 12 hr | 8 | 4 (50.0) | 7 | 2 (28.6) |
| 16 hr | 8 | 3 (37.5) | 7 | 2 (28.6) |
| 20 hr | 8 | 0 (0.0) | 7 | 0 (0.0) |
| 24 hr | 8 | 0 (0.0) | 7 | 0 (0.0) |
| Any time point | 8 | 5 (62.5) | 7 | 3 (42.9) |
|  |  |  |  |  |
| Age group: >=18 |  |  |  |  |
| Number and percentage of subjects with blood ammonia levels > ULN |  |  |  |  |
| 0 hr (pre-dose) | 6 | 1 (16.7) | 6 | 1 (16.7) |
| 4 hr | 6 | 1 (16.7) | 6 | 0 (0.0) |
| 8 hr | 6 | 1 (16.7) | 6 | 1 (16.7) |
| 12 hr | 6 | 2 (33.3) | 6 | 3 (50.0) |
| 16 hr | 6 | 2 (33.3) | 6 | 3 (50.0) |
| 20 hr | 6 | 1 (16.7) | 6 | 0 (0.0) |
| 24 hr | 6 | 1 (16.7) | 6 | 1 (16.7) |
| Any time point | 6 | 3 (50.0) | 6 | 5 (83.3) |

Abbreviations: GPB = glycerol phenylbutyrate; NaPBA = sodium phenylbutyrate; ULN = upper limit of normal. Measurement values are normalised ammonia results using the reference ULN of 35 µmol/L.
