# Supplementary material for: Long‐Term Efficacy and Safety of Glycerol Phenylbutyrate in Japanese Patients With Urea Cycle Disorders: Results From a Phase 3 Switch‐Over and 12‐Month Extension Study
Source: JIMD Rep. 2026 Jun 14;67(4):e70082. doi: 10.1002/jmd2.70082 (PMC13265243; doi:10.1002/jmd2.70082)
Supplement: Supplementary file 2 — Table S2: Glutamine levels (switch‐over phase). [file JMD2-67-e70082-s003.docx]

**Supplementary Table 2. Glutamine Levels(Switch-Over Phase)**

|  | n | Mean (S.D.) | Median | [Min, Max] |
| --- | --- | --- | --- | --- |
| Age group: All |  |  |  |  |
| NaPBA |  |  |  |  |
| Measurement value [μmol/L] |  |  |  |  |
| Day 7 | 16 | 703.35 (186.45) | 707.45 | [361.8, 1030.7] |
|  | | | | |
| GPB |  |  |  |  |
| Measurement value [μmol/L] |  |  |  |  |
| Day 14 | 15 | 603.99 (167.92) | 599.80 | [293.1, 975.0] |
|  | | | | |
| Change from Day 7 to Day 14 [μmol/L] |  |  |  |  |
| GPB (Day 14) - NaPBA (Day 7) | 15 | -96.01 (99.71) | -91.80 | [-278.5, 151.0] |
|  |  |  |  |  |
| Age group: 2<= <6 |  |  |  |  |
| NaPBA |  |  |  |  |
| Measurement value [μmol/L] |  |  |  |  |
| Day 7 | 2 | 876.10 (159.95) | 876.10 | [763.0, 989.2] |
|  |  |  |  |  |
| GPB |  |  |  |  |
| Measurement value [μmol/L] |  |  |  |  |
| Day 14 | 2 | 743.20 (101.82) | 743.20 | [671.2, 815.2] |
|  |  |  |  |  |
| Change from Day 7 to Day 14 [μmol/L] |  |  |  |  |
| GPB (Day 14) - NaPBA (Day 7) | 2 | -132.90 (58.12) | -132.90 | [-174.0, -91.8] |
|  |  |  |  |  |
| Age group: 6<= <18 |  |  |  |  |
| NaPBA |  |  |  |  |
| Measurement value [μmol/L] |  |  |  |  |
| Day 7 | 8 | 722.96 (205.37) | 728.25 | [361.8, 1030.7] |
|  |  |  |  |  |
| GPB |  |  |  |  |
| Measurement value [μmol/L] |  |  |  |  |
| Day 14 | 7 | 620.44 (193.23) | 548.40 | [452.8, 975.0] |
|  |  |  |  |  |
| Change from Day 7 to Day 14 [μmol/L] |  |  |  |  |
| GPB (Day 14) - NaPBA (Day 7) | 7 | -98.14 (143.02) | -69.90 | [-278.5, 151.0] |
|  |  |  |  |  |
| Age group: >=18 |  |  |  |  |
| NaPBA |  |  |  |  |
| Measurement value [μmol/L] |  |  |  |  |
| Day 7 | 6 | 619.62 (138.44) | 646.35 | [416.4, 792.8] |
|  |  |  |  |  |
| GPB |  |  |  |  |
| Measurement value [μmol/L] |  |  |  |  |
| Day 14 | 6 | 538.40 (137.04) | 590.90 | [293.1, 670.5] |
|  |  |  |  |  |
| Change from Day 7 to Day 14 [μmol/L] |  |  |  |  |
| GPB (Day 14) - NaPBA (Day 7) | 6 | -81.22 (42.42) | -83.70 | [-123.3, -34.0] |

Abbreviations: GPB = glycerol phenylbutyrate; Max = maximum; Min = minimum; NaPBA = sodium phenylbutyrate; S.D. = standard deviation.
