# Supplementary material for: Long‐Term Efficacy and Safety of Glycerol Phenylbutyrate in Japanese Patients With Urea Cycle Disorders: Results From a Phase 3 Switch‐Over and 12‐Month Extension Study
Source: JIMD Rep. 2026 Jun 14;67(4):e70082. doi: 10.1002/jmd2.70082 (PMC13265243; doi:10.1002/jmd2.70082)
Supplement: Supplementary file 3 — Table S3: Mean ammonia values during the extension phase. [file JMD2-67-e70082-s005.docx]

**Supplementary Table 3. Mean Ammonia Values During the Extension Phase**

|  | n | Mean (S.D.) | Median | [Min, Max] |
| --- | --- | --- | --- | --- |
| Age group: All |  |  |  |  |
| Measurement value [μmol/L] |  |  |  |  |
| Month 0 (Baseline) | 15 | 22.603 (7.709) | 22.990 | [9.55, 42.95] |
| Week 1 | 0 | - (-) | - | [- , -] |
| Month 1 | 14 | 20.970 (13.105) | 17.765 | [8.48, 58.33] |
| Month 2 | 14 | 18.873 (8.589) | 19.469 | [6.36, 33.41] |
| Month 3 | 14 | 22.779 (17.247) | 14.318 | [10.64, 73.18] |
| Month 6 | 13 | 20.183 (8.185) | 19.621 | [13.26, 44.55] |
| Month 9 | 14 | 28.055 (17.319) | 22.273 | [12.73, 74.77] |
| Month 12 | 14 | 20.969 (7.555) | 19.091 | [6.89, 37.65] |
| Completion/Discontinuation | 0 | - (-) | - | [- , -] |
| Age group: 2<= <6 |  |  |  |  |
| Measurement value [μmol/L] |  |  |  |  |
| Month 0 (Baseline) | 2 | 20.231 (1.387) | 20.231 | [19.25, 21.21] |
| Week 1 | 0 | - (-) | - | [- , -] |
| Month 1 | 2 | 20.311 (0.975) | 20.311 | [19.62, 21.00] |
| Month 2 | 2 | 21.855 (0.591) | 21.855 | [21.44, 22.27] |
| Month 3 | 2 | 22.617 (4.762) | 22.617 | [19.25, 25.98] |
| Month 6 | 2 | 23.009 (3.459) | 23.009 | [20.56, 25.45] |
| Month 9 | 2 | 24.865 (6.084) | 24.865 | [20.56, 29.17] |
| Month 12 | 2 | 20.152 (3.750) | 20.152 | [17.50, 22.80] |
| Completion/Discontinuation | 0 | - (-) | - | [- , -] |
| Age group: 6<= <18 |  |  |  |  |
| Measurement value [μmol/L] |  |  |  |  |
| Month 0 (Baseline) | 7 | 18.587 (6.153) | 21.212 | [9.55, 23.86] |
| Week 1 | 0 | - (-) | - | [- , -] |
| Month 1 | 7 | 16.439 (9.754) | 13.258 | [8.48, 37.65] |
| Month 2 | 7 | 17.259 (8.566) | 17.500 | [6.36, 26.52] |
| Month 3 | 7 | 24.020 (22.651) | 14.318 | [10.64, 73.18] |
| Month 6 | 6 | 17.146 (3.176) | 17.235 | [13.26, 20.68] |
| Month 9 | 7 | 35.143 (21.786) | 35.000 | [12.73, 74.77] |
| Month 12 | 7 | 18.757 (7.603) | 17.500 | [6.89, 30.54] |
| Completion/Discontinuation | 0 | - (-) | - | [- , -] |
| Age group: >=18 |  |  |  |  |
| Measurement value [μmol/L] |  |  |  |  |
| Month 0 (Baseline) | 6 | 28.078 (7.733) | 24.129 | [23.33, 42.95] |
| Week 1 | 0 | - (-) | - | [- , -] |
| Month 1 | 5 | 27.576 (18.015) | 21.212 | [12.73, 58.33] |
| Month 2 | 5 | 19.939 (10.917) | 12.727 | [11.14, 33.41] |
| Month 3 | 5 | 21.106 (13.610) | 14.318 | [11.14, 44.55] |
| Month 6 | 5 | 22.697 (12.635) | 15.909 | [14.85, 44.55] |
| Month 9 | 5 | 19.409 (8.195) | 14.318 | [13.26, 32.35] |
| Month 12 | 5 | 24.394 (8.468) | 19.621 | [18.03, 37.65] |
| Completion/Discontinuation | 0 | - (-) | - | [- , -] |

Abbreviations: Max = maximum; Min = minimum; S.D. = standard deviation; ULN = upper limit of normal. Measurement values are normalised ammonia results using the reference ULN of 35 µmol/L.
