# Supplementary material for: Long‐Term Efficacy and Safety of Glycerol Phenylbutyrate in Japanese Patients With Urea Cycle Disorders: Results From a Phase 3 Switch‐Over and 12‐Month Extension Study
Source: JIMD Rep. 2026 Jun 14;67(4):e70082. doi: 10.1002/jmd2.70082 (PMC13265243; doi:10.1002/jmd2.70082)
Supplement: Supplementary file 4 — Table S4: Summary of PK parameters of PBA, PAA, and PAGN (switch‐over, intent‐to‐treat population). [file JMD2-67-e70082-s007.docx]

**Supplementary Table 4. Summary of PK Parameters of PBA, PAA, and PAGN (Switch-Over, Intent-to-treat Population)**

|  | NaPBA (Day 7) | | | | | |  | GPB (Day 14) | | | | | |
| --- | --- | --- | --- | --- | --- | --- | --- | --- | --- | --- | --- | --- | --- |
|  | AUC_0-24_ [μg·hr/mL] | C_max_ [μg/mL] | C_min_ [μg/mL] | %Fluctuation | T_max_ [hr] | CL_ss_/F [mL/hr] |  | AUC_0-24_ [μg·hr/mL] | C_max_ [μg/mL] | C_min_ [μg/mL] | %Fluctuation | T_max_ [hr] | CL_ss_/F [mL/hr] |
| PAA |  |  |  |  |  |  |  |  |  |  |  |  |  |
| n | 16 | 16 | 16 | 16 | 16 | 16 |  | 15 | 15 | 15 | 15 | 15 | 15 |
| Mean (S.D.) | 984 (969) | 73.3 (61.9) | 7.05 (17.9) | 214.25 (71.65) | 10.37 (3.38) | 21638.6 (23774.6) |  | 1420 (2060) | 86.9 (92.4) | 32.3 (74.4) | 192.02 (92.35) | 10.13 (3.68) | 24468.9 (36300.2) |
| CV (%) | 98.5 | 84.5 | 253.8 | 33.4 | 32.6 | 109.9 |  | 145.0 | 106.4 | 230.6 | 48.1 | 36.4 | 148.4 |
| Median | 516 | 46.5 | 0.629 | 204.42 | 11.77 | 9719.4 |  | 539 | 49.5 | 0.842 | 175.88 | 7.72 | 12072.2 |
| [Min, Max] | [37.0, 2500  ] | [3.85, 190] | [0.00, 69.2] | [72.7, 348.9] | [3.6, 16.3] | [3817, 83151] |  | [17.5, 7510] | [1.69, 344] | [0.00, 261] | [26.2, 336.4] | [7.5, 16.1] | [1183, 144775] |
| Geometric Mean | 510 | 46.0 | - | 201.03 | 9.77 | 12846.0 |  | 591 | 49.7 | - | 160.93 | 9.59 | 12009.1 |
| Geometric CV (%) | 228.4 | 163.9 | - | 40.9 | 39.0 | 138.9 |  | 301.2 | 202.1 | - | 82.9 | 33.9 | 194.8 |
|  | | | | | | | | | | | | | |
| PBA |  |  |  |  |  |  |  |  |  |  |  |  |  |
| n | 16 | 16 | 16 | 16 | 16 | 16 |  | 15 | 15 | 15 | 15 | 15 | 15 |
| Mean (S.D.) | 425 (396) | 62.1 (58.8) | 0.499 (0.773) | 365.96 (99.48) | 11.36 (1.50) | 75177.9 (112124.9) |  | 470 (417) | 59.7 (51.7) | 0.702 (0.741) | 296.53 (77.83) | 9.82 (2.00) | 39969.0 (45410.0) |
| CV (%) | 93.2 | 94.7 | 154.9 | 27.2 | 13.2 | 149.1 |  | 88.7 | 86.6 | 105.6 | 26.2 | 20.3 | 113.6 |
| Median | 319 | 51.3 | 0.00 | 372.04 | 11.80 | 27890.9 |  | 417 | 50.9 | 0.640 | 278.35 | 9.78 | 21995.6 |
| [Min, Max] | [6.05, 1280] | [0.754, 224] | [0.00, 2.40] | [230.4, 569.7] | [7.5, 12.3] | [3422, 436302] |  | [67.9, 1620] | [6.29, 180] | [0.00, 2.13] | [176.3, 487.1] | [7.5, 12.0] | [7098, 180232] |
| Geometric Mean | 229 | 34.5 | - | 353.69 | 11.24 | 34434.1 |  | 322 | 39.7 | - | 287.35 | 9.63 | 26606.0 |
| Geometric CV (%) | 250.5 | 247.9 | - | 27.5 | 15.8 | 200.2 |  | 120.3 | 132.4 | - | 26.5 | 20.9 | 106.9 |
|  | | | | | | | | | | | | | |
| PAGN |  |  |  |  |  |  |  |  |  |  |  |  |  |
| n | 16 | 16 | 16 | 16 | 16 | 16 |  | 15 | 15 | 15 | 15 | 15 | 15 |
| Mean (S.D.) | 741 (389) | 51.7 (22.9) | 6.10 (7.21) | 154.76 (39.12) | 8.56 (2.79) | 21405.2 (9380.6) |  | 836 (518) | 53.3 (23.9) | 16.1 (22.6) | 127.95 (55.14) | 8.00 (3.15) | 20209.0 (6809.7) |
| CV (%) | 52.6 | 44.3 | 118.0 | 25.3 | 32.6 | 43.8 |  | 61.9 | 45.0 | 140.5 | 43.1 | 39.3 | 33.7 |
| Median | 705 | 48.9 | 3.55 | 160.52 | 7.83 | 21953.0 |  | 736 | 49.5 | 6.48 | 135.01 | 7.67 | 21765.2 |
| [Min, Max] | [288, 1780] | [25.0, 107] | [0.00, 22.0] | [80.1, 206.4] | [3.6, 12.3] | [6333, 44303] |  | [339, 2320] | [23.9, 110] | [1.06, 84.9] | [25.6, 221.4] | [0.0, 15.7] | [7429, 28061] |
| Geometric Mean | 662 | 47.6 | - | 149.46 | 8.06 | 19192.4 |  | 734 | 49.2 | 7.22 | 113.57 | - | 18756.3 |
| Geometric CV (%) | 50.9 | 43.2 | - | 29.0 | 39.2 | 55.8 |  | 52.8 | 41.9 | 215.2 | 60.9 | - | 45.9 |

Abbreviations: AUC_0-24_ = area under the concentration vs. time curve from hour 0 to 24 hours; CL_ss_/F = apparent clearance at steady state; C_max_ = maximum concentration of the drug after dosing; C_min_ = minimum concentration of the drug after dosing; CV = coefficient of variation; GPB = glycerol phenylbutyrate; Max = maximum; Min = minimum; NaPBA = sodium phenylbutyrate; PAA = phenylacetate; PAGN = phenylacetylglutamine; PBA = phenylbutyrate; PK = pharmacokinetic; S.D. = standard deviation; Tmax = time to Cmax.
