# Supplementary material for: Long‐Term Efficacy and Safety of Glycerol Phenylbutyrate in Japanese Patients With Urea Cycle Disorders: Results From a Phase 3 Switch‐Over and 12‐Month Extension Study
Source: JIMD Rep. 2026 Jun 14;67(4):e70082. doi: 10.1002/jmd2.70082 (PMC13265243; doi:10.1002/jmd2.70082)
Supplement: Supplementary file 5 — Table S5: Summary of PK parameters of PAA, PBA, and PAGN by age (switch‐over, intent‐to‐treat population). Table S5a. Age group: 0 ≤ < 2. Table S5b. Age group: 2 ≤ < 6. Table S5c. Age group: 6 ≤ < 18. Table S5d. Age group: ≥ 18. [file JMD2-67-e70082-s004.docx]

**Supplementary Table 5. Summary of PK Parameters of PAA, PBA, and PAGN by Age (Switch-Over, Intent-to-treat Population)**

**Supplementary Table 5a. Age group : 0<= <2**

|  | NaPBA (Day 7) | | | | | |  | GPB (Day 14) | | | | | |
| --- | --- | --- | --- | --- | --- | --- | --- | --- | --- | --- | --- | --- | --- |
|  | AUC_0-24_ [μg·hr/mL] | C_max_ [μg/mL] | C_min_ [μg/mL] | %Fluctuation | T_max_ [hr] | CL_ss_/F [mL/hr] |  | AUC_0-24_ [μg·hr/mL] | C_max_ [μg/mL] | C_min_ [μg/mL] | %Fluctuation | T_max_ [hr] | CL_ss_/F [mL/hr] |
| PAA |  |  |  |  |  |  |  |  |  |  |  |  |  |
| n | 0 | 0 | 0 | 0 | 0 | 0 |  | 0 | 0 | 0 | 0 | 0 | 0 |
| Mean (S.D.) | - (-) | - (-) | - (-) | - (-) | - (-) | - (-) |  | - (-) | - (-) | - (-) | - (-) | - (-) | - (-) |
| CV (%) | - | - | - | - | - | - |  | - | - | - | - | - | - |
| Median | - | - | - | - | - | - |  | - | - | - | - | - | - |
| [Min, Max] | [- , -] | [- , -] | [- , -] | [- , -] | [- , -] | [- , -] |  | [- , -] | [- , -] | [- , -] | [- , -] | [- , -] | [- , -] |
| Geometric Mean | - | - | - | - | - | - |  | - | - | - | - | - | - |
| Geometric CV (%) | - | - | - | - | - | - |  | - | - | - | - | - | - |
|  | | | | | | | | | | | | | |
| PBA | 0 | 0 | 0 | 0 | 0 | 0 |  | 0 | 0 | 0 | 0 | 0 | 0 |
| n | - (-) | - (-) | - (-) | - (-) | - (-) | - (-) |  | - (-) | - (-) | - (-) | - (-) | - (-) | - (-) |
| Mean (S.D.) | - | - | - | - | - | - |  | - | - | - | - | - | - |
| CV (%) | - | - | - | - | - | - |  | - | - | - | - | - | - |
| Median | [- , -] | [- , -] | [- , -] | [- , -] | [- , -] | [- , -] |  | [- , -] | [- , -] | [- , -] | [- , -] | [- , -] | [- , -] |
| [Min, Max] | - | - | - | - | - | - |  | - | - | - | - | - | - |
| Geometric Mean | - | - | - | - | - | - |  | - | - | - | - | - | - |
| Geometric CV (%) |  |  |  |  |  |  |  |  |  |  |  |  |  |
|  | | | | | | | | | | | | | |
| PAGN | - (-) | - (-) | - (-) | - (-) | - (-) | - (-) |  | - (-) | - (-) | - (-) | - (-) | - (-) | - (-) |
| n | - | - | - | - | - | - |  | - | - | - | - | - | - |
| Mean (S.D.) | - | - | - | - | - | - |  | - | - | - | - | - | - |
| CV (%) | [- , -] | [- , -] | [- , -] | [- , -] | [- , -] | [- , -] |  | [- , -] | [- , -] | [- , -] | [- , -] | [- , -] | [- , -] |
| Median | - | - | - | - | - | - |  | - | - | - | - | - | - |
| [Min, Max] | - | - | - | - | - | - |  | - | - | - | - | - | - |
| Geometric Mean | 0 | 0 | 0 | 0 | 0 | 0 |  | 0 | 0 | 0 | 0 | 0 | 0 |
| Geometric CV (%) | - (-) | - (-) | - (-) | - (-) | - (-) | - (-) |  | - (-) | - (-) | - (-) | - (-) | - (-) | - (-) |

Abbreviations: AUC_0-24_ = area under the concentration vs. time curve from hour 0 to 24 hours; CL_ss_/F = apparent clearance at steady state; C_max_ = maximum concentration of the drug after dosing; C_min_ = minimum concentration of the drug after dosing; CV = coefficient of variation; GPB = glycerol phenylbutyrate; Max = maximum; Min = minimum; NaPBA = sodium phenylbutyrate; PAA = phenylacetate; PAGN = phenylacetylglutamine; PBA = phenylbutyrate; PK = pharmacokinetic; S.D. = standard deviation; Tmax = time to Cmax.

**Supplementary Table 5b. Age group : 2<= <6**

|  | NaPBA (Day 7) | | | | | |  | GPB (Day 14) | | | | | |
| --- | --- | --- | --- | --- | --- | --- | --- | --- | --- | --- | --- | --- | --- |
|  | AUC_0-24_ [μg·hr/mL] | C_max_ [μg/mL] | C_min_ [μg/mL] | %Fluctuation | T_max_ [hr] | CL_ss_/F [mL/hr] |  | AUC_0-24_ [μg·hr/mL] | C_max_ [μg/mL] | C_min_ [μg/mL] | %Fluctuation | T_max_ [hr] | CL_ss_/F [mL/hr] |
| PAA |  |  |  |  |  |  |  |  |  |  |  |  |  |
| n | 2 | 2 | 2 | 2 | 2 | 2 |  | 2 | 2 | 2 | 2 | 2 | 2 |
| Mean (S.D.) | 273 (334) | 22.5 (26.4) | 0.348 (0.492) | 218.27 (43.35) | 9.79 (3.12) | 32274.1  (37992.1) |  | 781 (1080) | 56.3 (77.3) | 4.78 (6.75) | 191.50 (51.46) | 9.70 (2.92) | 73620.5  (100627.8) |
| CV (%) | 122.3 | 117.2 | 141.4 | 19.9 | 31.9 | 117.7 |  | 138.2 | 137.2 | 141.4 | 26.9 | 30.1 | 136.7 |
| Median | 273 | 22.5 | 0.348 | 218.27 | 9.79 | 32274.1 |  | 781 | 56.3 | 4.78 | 191.50 | 9.70 | 73620.5 |
| [Min, Max] | [37.0 , 509] | [3.85 , 41.2] | [0.00 , 0.696] | [187.6 , 248.9] | [7.6 , 12.0] | [5410 , 59139] |  | [17.5 , 1540] | [1.69 , 111] | [0.00 , 9.55] | [155.1 , 227.9] | [7.6 , 11.8] | [2466 ,  144775] |
| Geometric Mean | 137 | 12.6 | - | 216.11 | 9.54 | 17886.1 |  | 164 | 13.7 | - | 188.01 | 9.48 | 18894.3 |
| Geometric CV (%) | 548.7 | 395.0 | - | 20.2 | 33.3 | 405.8 |  | 15040.7 | 7968.3 | - | 27.7 | 31.3 | 6321.1 |
|  | | | | | | | | | | | | | |
| PBA |  |  |  |  |  |  |  |  |  |  |  |  |  |
| n | 2 | 2 | 2 | 2 | 2 | 2 |  | 2 | 2 | 2 | 2 | 2 | 2 |
| Mean (S.D.) | 489 (683) | 56.9 (79.4) | 0.278 (0.393) | 285.72 (17.69) | 11.88 (0.18) | 219862.2  (306092.6) |  | 380 (377) | 40.3 (44.0) | 0.675 (0.955) | 223.50 (45.47) | 9.58 (2.90) | 17057.5  (14084.8) |
| CV (%) | 139.7 | 139.5 | 141.4 | 6.2 | 1.5 | 139.2 |  | 99.3 | 109.2 | 141.4 | 20.3 | 30.3 | 82.6 |
| Median | 489 | 56.9 | 0.278 | 285.72 | 11.88 | 219862.2 |  | 380 | 40.3 | 0.675 | 223.50 | 9.58 | 17057.5 |
| [Min, Max] | [6.05 , 971] | [0.754 , 113] | [0.00 , 0.556] | [273.2 , 298.2] | [11.8 , 12.0] | [3422 ,  436302] |  | [113 , 647] | [9.17 , 71.4] | [0.00 , 1.35] | [191.3 , 255.7] | [7.5 , 11.6] | [7098 , 27017] |
| Geometric Mean | 76.7 | 9.23 | - | 285.45 | 11.87 | 38639.9 |  | 271 | 25.6 | - | 221.17 | 9.36 | 13848.0 |
| Geometric CV (%) | 63117.4 | 53080.7 | - | 6.2 | 1.5 | 35638.7 |  | 188.7 | 268.6 | - | 20.7 | 31.5 | 120.1 |
|  | | | | | | | | | | | | | |
| PAGN |  |  |  |  |  |  |  |  |  |  |  |  |  |
| n | 2 | 2 | 2 | 2 | 2 | 2 |  | 2 | 2 | 2 | 2 | 2 | 2 |
| Mean (S.D.) | 739 (150) | 58.8 (5.94) | 1.92 (2.71) | 185.84 (29.05) | 9.79 (3.12) | 6521.7 (267.5) |  | 700 (340) | 44.2 (14.5) | 13.3 (15.0) | 118.50 (59.36) | 7.58 (0.07) | 9283.3  (2020.9) |
| CV (%) | 20.2 | 10.1 | 141.4 | 15.6 | 31.9 | 4.1 |  | 48.6 | 32.8 | 112.9 | 50.1 | 0.9 | 21.8 |
| Median | 739 | 58.8 | 1.92 | 185.84 | 9.79 | 6521.7 |  | 700 | 44.2 | 13.3 | 118.50 | 7.58 | 9283.3 |
| [Min, Max] | [633 , 845] | [54.6 , 63.0] | [0.00 , 3.83] | [165.3 , 206.4] | [7.6 , 12.0] | [6333 , 6711] |  | [460 , 941] | [33.9 , 54.4] | [2.68 , 23.9] | [76.5 , 160.5] | [7.5 , 7.6] | [7854 , 10712] |
| Geometric Mean | 731 | 58.6 | - | 184.70 | 9.54 | 6519.0 |  | 658 | 42.9 | 8.00 | 110.82 | 7.58 | 9172.6 |
| Geometric CV (%) | 20.6 | 10.1 | - | 15.8 | 33.3 | 4.1 |  | 54.0 | 34.4 | 315.5 | 56.2 | 0.9 | 22.2 |

Abbreviations: AUC_0-24_ = area under the concentration vs. time curve from hour 0 to 24 hours; CL_ss_/F = apparent clearance at steady state; C_max_ = maximum concentration of the drug after dosing; C_min_ = minimum concentration of the drug after dosing; CV = coefficient of variation; GPB = glycerol phenylbutyrate; Max = maximum; Min = minimum; NaPBA = sodium phenylbutyrate; PAA = phenylacetate; PAGN = phenylacetylglutamine; PBA = phenylbutyrate; PK = pharmacokinetic; S.D. = standard deviation; Tmax = time to Cmax.

**Supplementary Table 5c. Age group : 6<= <18**

|  | NaPBA (Day 7) | | | | | |  | GPB (Day 14) | | | | | |
| --- | --- | --- | --- | --- | --- | --- | --- | --- | --- | --- | --- | --- | --- |
|  | AUC_0-24_ [μg·hr/mL] | C_max_ [μg/mL] | C_min_ [μg/mL] | %Fluctuation | T_max_ [hr] | CL_ss_/F [mL/hr] |  | AUC_0-24_ [μg·hr/mL] | C_max_ [μg/mL] | C_min_ [μg/mL] | %Fluctuation | T_max_ [hr] | CL_ss_/F [mL/hr] |
| PAA |  |  |  |  |  |  |  |  |  |  |  |  |  |
| n | 8 | 8 | 8 | 8 | 8 | 8 |  | 7 | 7 | 7 | 7 | 7 | 7 |
| Mean (S.D.) | 1370 (1020) | 101 (67.0) | 5.15 (9.22) | 210.92 (78.05) | 10.93 (3.65) | 14878.1  (16823.5) |  | 1890 (2550) | 113 (109) | 44.7 (96.2) | 178.84 (94.67) | 11.09 (4.31) | 11695.2  (10146.8) |
| CV (%) | 74.8 | 66.2 | 179.0 | 37.0 | 33.4 | 113.1 |  | 135.1 | 96.0 | 215.2 | 52.9 | 38.8 | 86.8 |
| Median | 1540 | 109 | 1.21 | 189.86 | 11.80 | 6904.4 |  | 1080 | 83.0 | 1.95 | 175.88 | 7.72 | 9365.7 |
| [Min, Max] | [153 , 2500] | [18.2 , 190] | [0.00 , 26.7] | [118.9 , 348.9] | [3.6 , 15.9] | [3817 , 47660] |  | [230 , 7510] | [25.7 , 344] | [0.00 , 261] | [26.2 , 310.2] | [7.6 , 15.8] | [1183 , 33162] |
| Geometric Mean | 866 | 75.9 | - | 198.98 | 10.15 | 9363.3 |  | 1020 | 82.3 | - | 145.51 | 10.40 | 8292.9 |
| Geometric CV (%) | 176.3 | 111.9 | - | 37.6 | 48.6 | 123.3 |  | 169.8 | 101.1 | - | 98.6 | 40.0 | 131.3 |
|  | | | | | | | | | | | | | |
| PBA |  |  |  |  |  |  |  |  |  |  |  |  |  |
| n | 8 | 8 | 8 | 8 | 8 | 8 |  | 7 | 7 | 7 | 7 | 7 | 7 |
| Mean (S.D.) | 405 (496) | 63.6 (78.5) | 0.687 (0.974) | 381.58  (101.07) | 10.82 (2.04) | 78090.5  (71883.6) |  | 419 (562) | 57.8 (68.6) | 0.751 (0.826) | 325.98 (96.12) | 10.41 (1.93) | 65162.7  (58082.6) |
| CV (%) | 122.3 | 123.4 | 141.9 | 26.5 | 18.8 | 92.1 |  | 134.4 | 118.7 | 110.0 | 29.5 | 18.6 | 89.1 |
| Median | 162 | 26.4 | 0.267 | 392.80 | 11.77 | 54346.6 |  | 154 | 19.2 | 0.640 | 332.65 | 11.80 | 54238.3 |
| [Min, Max] | [42.1 , 1280] | [6.93 , 224] | [0.00 , 2.40] | [230.4 , 528.3] | [7.5 , 12.3] | [9397 ,  209105] |  | [67.9 , 1620] | [6.29 , 180] | [0.00 , 2.13] | [176.3 , 487.1] | [7.7 , 12.0] | [10396 ,  180232] |
| Geometric Mean | 204 | 32.3 | - | 368.86 | 10.63 | 48052.1 |  | 226 | 30.9 | - | 313.05 | 10.25 | 45286.8 |
| Geometric CV (%) | 198.9 | 196.9 | - | 29.2 | 21.5 | 163.0 |  | 161.0 | 182.7 | - | 32.4 | 20.0 | 125.7 |
|  | | | | | | | | | | | | | |
| PAGN |  |  |  |  |  |  |  |  |  |  |  |  |  |
| n | 8 | 8 | 8 | 8 | 8 | 8 |  | 7 | 7 | 7 | 7 | 7 | 7 |
| Mean (S.D.) | 843 (522) | 55.1 (30.1) | 7.86 (8.52) | 143.94 (37.33) | 8.79 (3.78) | 23965.9  (10313.6) |  | 997 (717) | 59.8 (33.3) | 22.5 (31.2) | 118.97 (56.12) | 8.29 (4.78) | 21750.5  (7468.0) |
| CV (%) | 61.9 | 54.6 | 108.3 | 25.9 | 43.0 | 43.0 |  | 71.9 | 55.6 | 138.6 | 47.2 | 57.7 | 34.3 |
| Median | 763 | 48.9 | 4.79 | 150.53 | 9.64 | 21476.3 |  | 749 | 49.5 | 6.61 | 134.58 | 7.67 | 25145.4 |
| [Min, Max] | [288 , 1780] | [25.0 , 107] | [0.915 , 22.0] | [80.1 , 195.4] | [3.6 , 12.3] | [10907 ,  44303] |  | [339 , 2320] | [23.9 , 110] | [1.06 , 84.9] | [25.6 , 193.0] | [0.0 , 15.7] | [7429 , 28061] |
| Geometric Mean | 711 | 48.7 | 4.35 | 139.18 | 7.90 | 22154.8 |  | 818 | 52.6 | 8.33 | 102.30 | - | 20124.2 |
| Geometric CV (%) | 70.5 | 56.4 | 184.7 | 29.3 | 56.9 | 44.6 |  | 74.3 | 59.0 | 358.5 | 76.7 | - | 50.5 |

Abbreviations: AUC_0-24_ = area under the concentration vs. time curve from hour 0 to 24 hours; CL_ss_/F = apparent clearance at steady state; C_max_ = maximum concentration of the drug after dosing; C_min_ = minimum concentration of the drug after dosing; CV = coefficient of variation; GPB = glycerol phenylbutyrate; Max = maximum; Min = minimum; NaPBA = sodium phenylbutyrate; PAA = phenylacetate; PAGN = phenylacetylglutamine; PBA = phenylbutyrate; PK = pharmacokinetic; S.D. = standard deviation; Tmax = time to Cmax.

**Supplementary Table 5d. Age group : >=18**

|  | NaPBA (Day 7) | | | | | |  | GPB (Day 14) | | | | | |
| --- | --- | --- | --- | --- | --- | --- | --- | --- | --- | --- | --- | --- | --- |
|  | AUC_0-24_ [μg·hr/mL] | C_max_ [μg/mL] | C_min_ [μg/mL] | %Fluctuation | T_max_ [hr] | CL_ss_/F [mL/hr] |  | AUC_0-24_ [μg·hr/mL] | C_max_ [μg/mL] | C_min_ [μg/mL] | %Fluctuation | T_max_ [hr] | CL_ss_/F [mL/hr] |
| PAA |  |  |  |  |  |  |  |  |  |  |  |  |  |
| n | 6 | 6 | 6 | 6 | 6 | 6 |  | 6 | 6 | 6 | 6 | 6 | 6 |
| Mean (S.D.) | 709 (895) | 52.9 (47.8) | 11.8 (28.1) | 217.36 (80.38) | 9.81 (3.56) | 27107.3  (29266.3) |  | 1090 (1790) | 66.2 (81.7) | 26.9 (61.8) | 207.56  (109.83) | 9.16 (3.40) | 22987.7  (18531.1) |
| CV (%) | 126.3 | 90.4 | 238.0 | 37.0 | 36.3 | 108.0 |  | 165.0 | 123.4 | 229.8 | 52.9 | 37.1 | 80.6 |
| Median | 469 | 46.5 | 0.281 | 227.50 | 7.83 | 15255.6 |  | 463 | 37.2 | 0.421 | 194.49 | 7.77 | 18166.1 |
| [Min, Max] | [52.7 , 2500] | [6.32 , 144] | [0.00 , 69.2] | [72.7 , 289.1] | [7.6 , 16.3] | [4385 , 83151] |  | [92.7 , 4730] | [12.9 , 231] | [0.00 , 153] | [39.7 , 336.4] | [7.5 , 16.1] | [2412 , 54715] |
| Geometric Mean | 389 | 36.4 | - | 198.96 | 9.36 | 17537.5 |  | 476 | 42.5 | - | 171.86 | 8.77 | 15904.1 |
| Geometric CV (%) | 197.7 | 139.3 | - | 55.4 | 32.8 | 135.0 |  | 211.7 | 121.5 | - | 91.3 | 30.5 | 147.8 |
|  | | | | | | | | | | | | | |
| PBA |  |  |  |  |  |  |  |  |  |  |  |  |  |
| n | 6 | 6 | 6 | 6 | 6 | 6 |  | 6 | 6 | 6 | 6 | 6 | 6 |
| Mean (S.D.) | 429 (175) | 61.8 (21.6) | 0.322 (0.563) | 371.87  (110.99) | 11.90 (0.15) | 23066.4  (9877.9) |  | 561 (250) | 68.4 (34.7) | 0.653 (0.728) | 286.53 (44.64) | 9.21 (2.01) | 18213.5  (4388.5) |
| CV (%) | 40.8 | 34.9 | 175.1 | 29.8 | 1.3 | 42.8 |  | 44.7 | 50.7 | 111.4 | 15.6 | 21.8 | 24.1 |
| Median | 500 | 59.0 | 0.00 | 372.04 | 11.91 | 21448.3 |  | 493 | 57.4 | 0.540 | 276.01 | 8.13 | 17198.1 |
| [Min, Max] | [143 , 601] | [33.9 , 92.6] | [0.00 , 1.38] | [245.7 , 569.7] | [11.7 , 12.1] | [10977 ,  36842] |  | [252 , 941] | [29.0 , 127] | [0.00 , 1.50] | [234.9 , 355.7] | [7.6 , 11.9] | [13966 ,  24290] |
| Geometric Mean | 387 | 58.6 | - | 359.19 | 11.90 | 21249.2 |  | 514 | 61.5 | - | 283.73 | 9.04 | 17783.6 |
| Geometric CV (%) | 59.3 | 37.7 | - | 29.1 | 1.3 | 47.8 |  | 49.3 | 54.3 | - | 15.3 | 21.0 | 24.2 |
|  | | | | | | | | | | | | | |
| PAGN |  |  |  |  |  |  |  |  |  |  |  |  |  |
| n | 6 | 6 | 6 | 6 | 6 | 6 |  | 6 | 6 | 6 | 6 | 6 | 6 |
| Mean (S.D.) | 604 (172) | 45.0 (14.4) | 5.16 (6.30) | 158.83 (43.34) | 7.83 (0.25) | 22952.2  (3617.4) |  | 694 (205) | 48.7 (10.8) | 9.58 (10.5) | 141.57 (60.49) | 7.81 (0.28) | 22052.6  (2635.6) |
| CV (%) | 28.5 | 32.0 | 122.1 | 27.3 | 3.2 | 15.8 |  | 29.6 | 22.3 | 109.3 | 42.7 | 3.6 | 12.0 |
| Median | 589 | 40.5 | 2.80 | 166.72 | 7.83 | 22282.0 |  | 643 | 52.3 | 4.64 | 149.83 | 7.77 | 21711.4 |
| [Min, Max] | [381 , 834] | [30.8 , 67.1] | [1.82 , 17.9] | [90.2 , 204.2] | [7.6 , 8.1] | [17830 ,  28646] |  | [505 , 1050] | [33.6 , 59.8] | [2.23 , 28.5] | [63.3 , 221.4] | [7.5 , 8.3] | [19492 ,  26876] |
| Geometric Mean | 583 | 43.1 | 3.45 | 153.16 | 7.82 | 22715.8 |  | 671 | 47.6 | 5.91 | 129.35 | 7.81 | 21929.6 |
| Geometric CV (%) | 30.1 | 31.7 | 105.4 | 31.5 | 3.2 | 15.9 |  | 28.4 | 24.2 | 144.3 | 51.7 | 3.6 | 11.4 |

Abbreviations: AUC_0-24_ = area under the concentration vs. time curve from hour 0 to 24 hours; CL_ss_/F = apparent clearance at steady state; C_max_ = maximum concentration of the drug after dosing; C_min_ = minimum concentration of the drug after dosing; CV = coefficient of variation; GPB = glycerol phenylbutyrate; Max = maximum; Min = minimum; NaPBA = sodium phenylbutyrate; PAA = phenylacetate; PAGN = phenylacetylglutamine; PBA = phenylbutyrate; PK = pharmacokinetic; S.D. = standard deviation; Tmax = time to Cmax.
