# Supplementary material for: Long‐Term Efficacy and Safety of Glycerol Phenylbutyrate in Japanese Patients With Urea Cycle Disorders: Results From a Phase 3 Switch‐Over and 12‐Month Extension Study
Source: JIMD Rep. 2026 Jun 14;67(4):e70082. doi: 10.1002/jmd2.70082 (PMC13265243; doi:10.1002/jmd2.70082)
Supplement: Supplementary file 6 — Data S1: Plain language summary in Japanese. [file JMD2-67-e70082-s002.pdf]

プレーンランゲージサマリー

尿素サイクル異常症の日本人患者におけるフェニル酪酸グリセロールの長期使用

和田 陽一[1]、古城 真秀子[2]、鹿島田 健一[3]、濱崎 考史[4]、入月 浩美[5]、市本 景子[6]、垣内 俊彦[7]、松本 志郎[8]、渡邊 順子[9]、小野 知穂 [10]、清水 貴子 [10]、古川 宏郁 [10]、中村 公俊[8]

[1]東北大学病院、[2]岡山医療センター、[3]国立成育医療研究センター、[4]大阪公立大学医学部付属病院、[5]新潟大学医歯学総合病院、[6]千葉県こども病院、[7]佐賀大学医学部附属病院、[8]熊本大学病院、[9]久留米大学医学部附属病院、[10] 株式会社オーファンパシフィック

要約

この研究は、日本人の尿素サイクル異常症（UCD）患者さんを対象にフェニル酪酸ナトリウム（NaPBA）からフェニル酪酸グリセロール（GPB）に治療を切り替えたときの長期間の効果と安全性について調べたものです。

私たちの体では、食べ物に含まれるタンパク質を分解する際に老廃物として窒素をつくります。窒素は体の中でアンモニアに変化し、アンモニアが体内にたまると有害です。健康な人では、肝臓の「尿素サイクル」という仕組みによってアンモニアは害の少ない尿素に変わり、尿として体外に排出されます。UCD の患者さんでは、この仕組みがうまく働かないため、血液中にアンモニアがたまりやすくなります。体内のアンモニア濃度が高くなると、特に脳に悪い影響を与え、吐き気、混乱、意識を失うなどの症状が起こることがあります。

NaPBA は、尿素サイクルという仕組みを使わずに別の方法で、体の中のアンモニア濃度を下げる薬です。しかし、味がよくないこと、1 回に飲む量が多いこと、さらにナトリウムを多く含むといったことから、長く使い続けるのが困難な患者さんもいます。GPB は、こうした NaPBA の課題を改善するために開発された薬です。GPB はナトリウムを含まず、少ない量で服用でき、味もにおいもほとんどないという特徴があります。

この研究には、小児 10 名と成人 7 名の患者さんが参加しました。最初の 7 日間は NaPBA を服用し、その後の 7 日間は GPB（同じ量のフェニル酪酸を含む）に切り替えて服用しました。その結果、24 時間にわたって測定した平均アンモニア量は、NaPBA で治療したときは 757 µmol･h/L であったのに対して、GPB に切替えた後は 627 µmol･h/L になりました。この切替え期間中に、NaPBA または GPB の治療に関連した副作用はありませんでした。NaPBA 治療中と GPB 治療中に、それぞれ 1 名が高アンモニア血症を発症し、研究を中止しました。14 名の患者さんは、引き続き GPB で治療する研究に参加し、NaPBA で治療していない 1 名の患者さんも研究に加わりました。12 ヵ月のデータ集計時点では、14 名が GPB の治療を受けていました。継続投与期間中のアンモニア値は正常範囲内に保たれていました。副作用は、吐き気と QT 延長の 2 件が報告されました。重篤な有害事象 1 件（ノロウイルスによる胃腸炎）と 2 件の高アンモニア血症は治療とは無関係でした。これらの結果から、GPB は NaPBA に代わる実用的かつ臨床的にも利点のある治療の選択肢であり、日本の UCD 患者さんにおいてもこれまでに海外で報告されている結果と同じ傾向が、日本人患者さんでも確認されました。

この資料はどのような方に役立ちますか？

この資料は、UCD の患者さんやその家族および介護をしている方に役立つ内容です。また、患者支援に係る方や医療従事者の方にとっても参考になります。

尿素サイクル異常症（UCD）とは？

尿素サイクルは、主に肝臓で働く体の仕組みで、体内で余分にたまった窒素を取り除く役割を担っています。この仕組みによって、有害なアンモニアを害の少ない尿素に変え、尿素は腎臓に運ばれてろ過され、最終的に尿として体外に排出されます。

尿素サイクルが正常に働くためには、6 種類の酵素と 2 種類の輸送タンパク（トランスポーター）が必要です。UCD の人は、これらのいずれかが生まれつき欠損している、あるいはうまく働かないことによって起こる、まれな遺伝性の病気です。

UCD の 8 つのタイプと関連する疾患：

疾患	酵素欠損
**アルギニン血症（ARG 欠損症）**	アルギナーゼ (ARG)
**CPS-1 欠損症**	カルバミルリン酸合成酵素 (CPS)
**シトルリン血症 I 型（ASS 欠損症）**	アルギニノコハク酸合成酵素 (ASS)
**OTC 欠損症**	オルニチントランスカルバミラーゼ (OTC)
**アルギニノコハク酸尿症（ASL 欠損症）**	アルギニノコハク酸リアーゼ (ASL)
**NAGS 欠損症**	N-アセチルグルタミン酸合成酵素 (NAGS)
疾患	トランスポーター欠損
**高オルニチン・高アンモニア・ホモシトルリン尿症（HHH）症候群**	オルニチン･シトルリンアンチポーター (ORNT1)
**シトルリン血症 II 型（シトリン欠損症）**	シトリン

プレーンランゲージサマリー

UCD では、血液中のアンモニア濃度が非常に高くなり「高アンモニア血症」という状態になることがあります。その他の症状として、吐き気や食欲不振、呼吸が速くなる、けいれん、意識がぼんやりする、行動がいつもと違う、発達の遅れなどがみられます。症状が重い場合には、生命に関わることもあります。多くは乳児期に発症しますが、大人になってから診断される方もいます。

UCD の長期的な治療には、体内にアンモニアや余分な窒素がたまらないように、タンパク質の摂取量を調整する食事療法が行われます。ただし、高アンモニア血症になることへの不安からタンパク質を避けたり、必要以上に控えてしまう方もいます。その結果、成長や筋肉を保つために必要な量のタンパク質が足りなくなってしまうことがあります。そのため、UCD の治療では、体内の余分な窒素を処理する薬が使われ、体の中の窒素の増えすぎを防いで、必要なタンパク質の量を保つことができるように助ける役割を果たします。

どのような治療が検討されましたか？

NaPBA は、UCD の長期治療に用いられている、体内の余分な窒素を処理する薬です。NaPBA は体内の窒素と結合して、尿として体外に排出するのを助けます。これは、尿素サイクルとは別の方法で、余分な窒素を体から取り除く仕組みです。NaPBA は不要な窒素を排出するのに役立ちますが、味がよくないこと、投与量が多いこと、ナトリウムを多く含むことなど、飲み続けることが負担になる人もいます。

GPB は、こうした NaPBA の課題を改善する目的で開発された薬で、フェニル酪酸（PBA）のプロドラッグです。GPB はナトリウムを含まず、少ない量で服用でき、味もないという特徴があります。

「プロドラッグ」とは、体の中で変化してからはじめて効果を発揮する薬のことです。

なぜこの研究が行われたのですか？

米国およびカナダで行われた臨床試験では、GPB の効果は少なくとも NaPBA と同等であり、安全性も確認されています。しかし、これまで日本人の UCD 患者さんを対象に GPB について調べた臨床試験は行われていませんでした。人種や遺伝的な背景の違いによって、治療の効果に違いがある可能性があるため、日本人患者さんを対象とした調査が必要とされていました。

誰がこの試験に参加しましたか？

この研究では、最初の 14 日間の「切替え期間」に 17 名の患者さんが参加しました。そのうち 2 名は高アンモニア血症のため途中で研究を中止しました。「継続投与期間」では 15 名が参加し（切替え期間からの継続した 14 名と新たに参加した 1 名）、12 か月後の時点で 14 名が GPB による治療を受けていました。

この研究に参加するためには、以下の条件を満たす必要がありました。

- ✓ 年齢や性別に関係なく、UCD と診断されていること
- ✓ 切替え期間に参加する場合は、研究開始前の少なくとも 1 週間、NaPBA を安定した用量で使用していること
- ✓ 研究を始める前の 1 週間以内に、安息香酸ナトリウムを使用していないこと

以下に該当する場合は、研究に参加できませんでした。

- × 研究開始時に血液中の血中アンモニア値が非常に高い場合
- × 研究開始前に高アンモニア血症による症状があった場合
- × 感染症にかかっている、もしくはアンモニアの代謝に影響する可能性のある他の病気がある場合
- × 研究に参加することで、健康上のリスクが高くなると考えられる検査結果や病状がある場合
- × 腎臓の働き、タンパク質の分解または血中アンモニア値に影響を与える可能性のある薬を使用しているか、使用する予定がある場合
- × これまでに不整脈がある場合
- × 過去にフェニル酪酸や関連する物質でアレルギー反応が起きたことがある場合
- × 過去に肝移植を受けたことがある場合

参加者の特徴

切替え期間

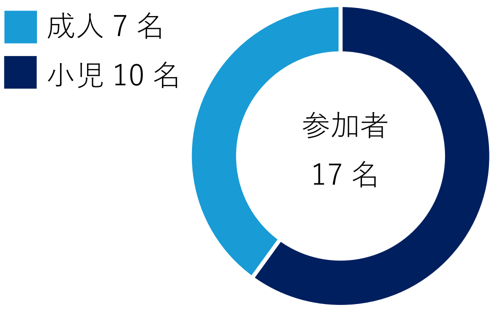

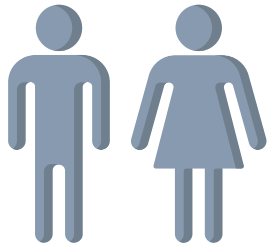

12 ヵ月継続投与期間

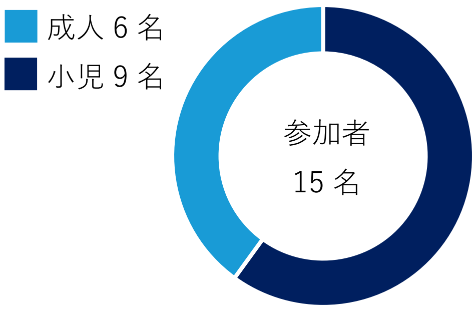

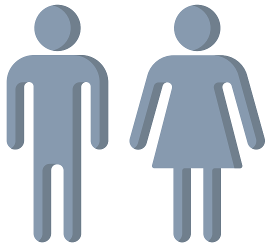

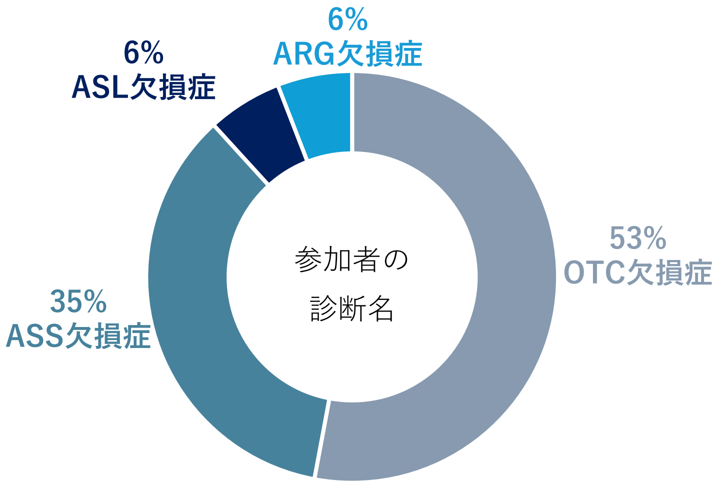

この研究ではどのようなことが行われましたか？

この研究は、第 3 相、非盲検、切替え試験と継続投与試験による研究でした。

第 3 相試験

臨床試験は、まず安全性を確認し、その後少人数の患者さんで効果を確かめたうえで、より多くの患者さんを対象に長期間の評価が行われます。

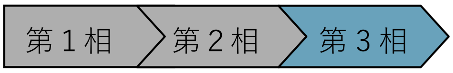

非盲検試験

この研究では、参加者と医師の双方が、どの薬を使っているかを知ったうえで行われました。

切替え期間と継続投与期間

この研究は、切替え期間と継続投与期間からなるデザインでした。研究の最初の段階の「切替え期間」では、17 名の参加者が最初の 7 日間は NaPBA を服用し、8 日目から 14 日目まで GPB を服用しました。いずれの薬も 1 日 3 回、食事とともにまたは食後すぐに服用しました。 切替え期間を完了した参加者は 15 名でした。

「継続投与期間」では、15 名（切替え期間から継続した 14 名と新たに参加した 1 名）が GPB による治療を受け、12 ヵ月時点では 14 名が GPB による治療を継続していました。

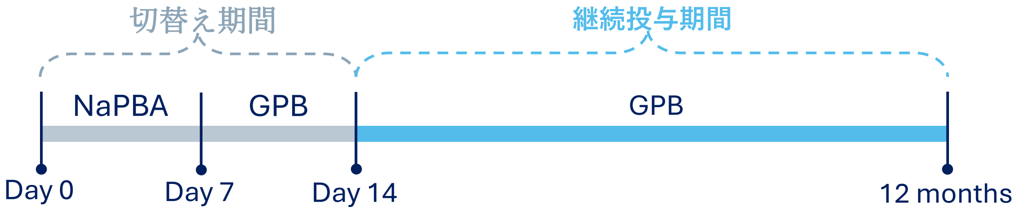

この研究では何が検討されましたか？

UCD の患者さんでは、アンモニアを分解するために必要な酵素や輸送タンパク（トランスポーター）が欠損しているため、血液中のアンモニアが正常値を超えて蓄積しやすい状態にあります。

プレーンランゲージサマリー

本研究では、「切替え期間」において NaPBA と GPB の効果を調べるため、7 日目（NaPBA の治療終了時）と 14 日目（GPB の治療終了時）に、それぞれ 24 時間にわたって血中アンモニア濃度を測定しました。さらに、GPB を長期使用したときの安全性や効果を調べるため、「継続投与期間」では 12 か月間にわたり、定期的に血中アンモニア濃度を測定しました。

この研究は複数の医療機関で行われたため、施設ごとの測定結果を公平に比較できるように、解析の前にアンモニア値を共通の基準範囲にそろえて調整しました。

主な研究結果は何でしたか？

この研究は 2023 年 4 月に開始され、日本全国の 9 つの医療機関で実施されました。

GPB に切り替えた後、24 時間にわたる血液中の平均アンモニア量は 627 μmol･h/L であり、NaPBA を使用したときの 757 μmol･h/L と比べて低くなりました。

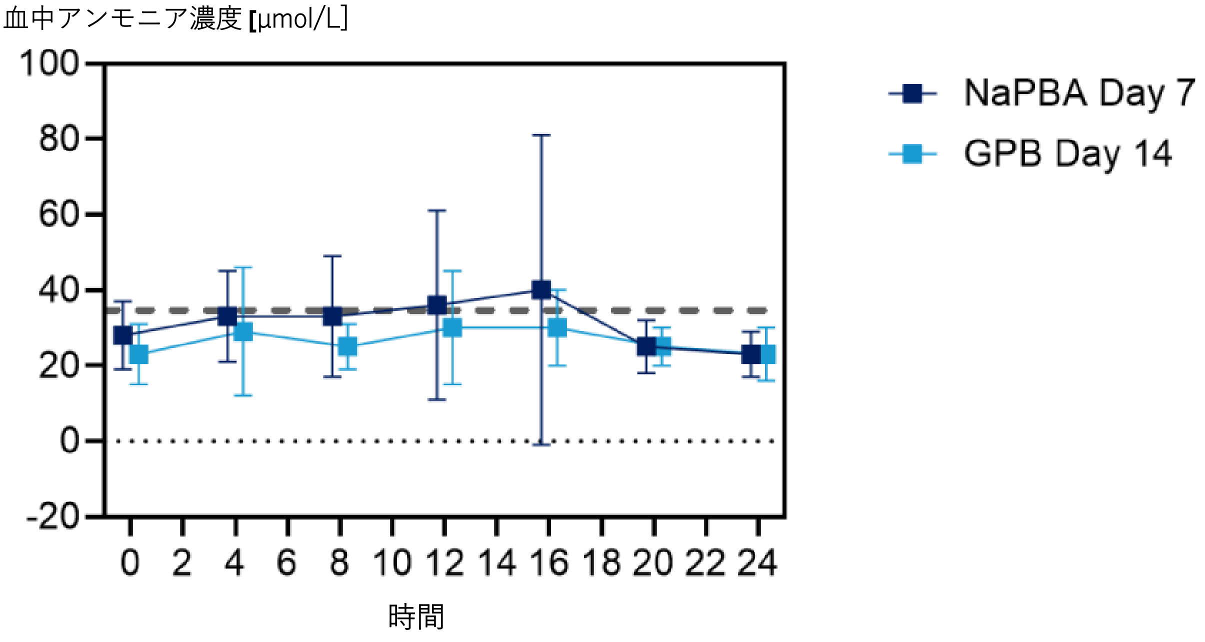

図 1. 24 時間にわたる平均血中アンモニア濃度（破線は標準化された正常上限値の 35 μmol/L を示す）

また、12 か月間の継続投与期間中も、平均の血中アンモニア濃度は正常範囲（11～35 μmol/L）内に保たれ、長期間安定してコントロールされることが示されました。

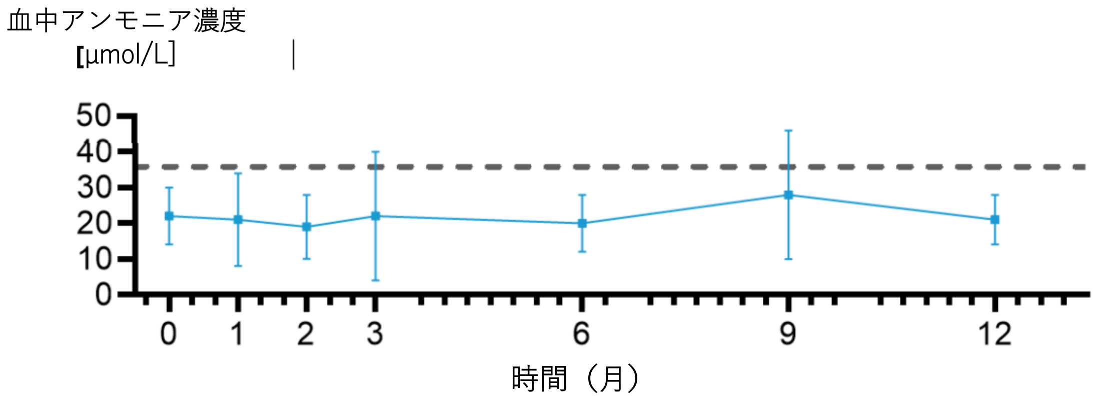

図 2. 12 か月間の追跡期間における平均血中アンモニア濃度の推移（破線は標準化された正常上限値 35 μmol/L を示す）

安全性に関する懸念はありましたか？

副作用の多くは軽度から中等度でした。切替え期間中には、NaPBA による治療中に 1 名、GPB による治療中に 1 名が高アンモニア血症を経験し、いずれの参加者も研究を中止しました。継続投与期間中には、副作用を理由に研究を中止した参加者はおらず、死亡例も報告されませんでした。

副作用として報告された 2 件（吐き気と QT 延長）は治療との関連があると判断されましたが、重篤な有害事象として報告された 1 件（ノロウイルスによる胃腸炎）と高アンモニア血症の 2 件は治療とは関係がないと判断されました。

研究期間全体を通して、血液検査の結果、心電図、アミノ酸の値において、治療を続けるうえで問題となる異常は見られませんでした。

この研究結果は何を意味しますか？

NaPBA は余分な窒素を除去する薬で、現在も日本において UCD の治療に最も広く使用されています。しかし本研究の結果から、医学的に必要と判断される場合には GPB への切り替えが可能であり、新たな安全性の問題が生じることはなく、アンモニアを良好にコントロールできることが示されました。

また、本研究の切替え期間の結果から、NaPBAによる治療が安定している患者は、GPBへ安全に移行できることが示されました。

GPBは服薬しやすく、服用する量が少なくなり、ナトリウムの負荷も少ないことから、患者が薬を適切に飲み続けやすく、その結果としてアンモニア濃度を長期にわたって良好に保つ可能性があると考えられます。

この治療は利用できますか？

GPBは、日本において2025年12月22日に、「ラヴィクティ®内用液1.1 g/mL」の製品名で製造販売承認を取得しました。

本研究の限界はありますか？

UCDは日本において非常にまれな疾患（出生約5万人に1人）であるため、この研究で検討された患者数は少数でした。また、本研究は非盲検試験であり、医療従事者および参加者の双方が、どの治療を受けているかを把握していました。

研究のスポンサーは誰ですか？

この研究は、株式会社オーファンパシフィックにより実施されました。著者の一部は、同社の社員です。

試験登録番号：jRCT2071220110
