# Supplementary material for: Long‐Term Efficacy and Safety of Glycerol Phenylbutyrate in Japanese Patients With Urea Cycle Disorders: Results From a Phase 3 Switch‐Over and 12‐Month Extension Study
Source: JIMD Rep. 2026 Jun 14;67(4):e70082. doi: 10.1002/jmd2.70082 (PMC13265243; doi:10.1002/jmd2.70082)
Supplement: Supplementary file 7 — Data S2: Plain language summary in English. [file JMD2-67-e70082-s006.pdf]

Long-term use of glycerol phenylbutyrate in Japanese patients with urea cycle disorders

Yoichi Wada[1], Mahoko Furujo[2], Kenichi Kashimada[3], Takashi Hamazaki[4], Hiromi Nyuzuki[5], Keiko Ichimoto[6], Toshihiko Kakiuchi[7], Shirou Matsumoto[8], Yoriko Watanabe[9], Chiho Ono[10], Takako Shimizu[10], Hiroiku Furukawa[10], Kimitoshi Nakamura[8]

[1]Tohoku University Hospital, [2]Okayama Medical Center, [3]National Center for Child Health & Development, [4]Osaka Metropolitan University, [5]Niigata University Medical & Dental Hospital, [6]Chiba Children's Hospital, [7]Saga University, [8]Kumamoto University, [9]Kurume University, [10]OrphanPacific Inc.

Summary

This plain language summary describes a long-term efficacy and safety study in Japanese patients with urea cycle disorders (UCDs) whose treatment was changed from sodium phenylbutyrate (NaPBA) to glycerol phenylbutyrate (GPB).

When the body breaks down protein from food, it produces nitrogen as a waste product. This nitrogen is carried in the body as ammonia, which is toxic if it builds up. In healthy people, the liver uses the urea cycle to convert ammonia into urea, a less harmful substance that is removed from the body in urine. In people with a UCD, this process does not work properly, causing ammonia to build up in the blood. High ammonia levels can be harmful, especially to the brain, and may cause symptoms such as nausea, confusion, or loss of consciousness.

Sodium phenylbutyrate helps reduce ammonia levels by providing an alternative way for nitrogen to be removed from the body without relying on the urea cycle. However, it has an unpleasant taste, requires large dosing volumes, and contains a high amount of sodium, which can make long-term treatment more difficult for some patients. Glycerol phenylbutyrate was developed to address these limitations. It does not contain sodium, it is more concentrated meaning volumes are smaller, and it is tasteless and odourless.

In this study comprising 10 children and 7 adults, participants received NaPBA for 7 days before being switched to an equivalent dose of GPB for 7 days. After switching to GPB, average ammonia exposure measured over 24 hours was 627 μmol·h/L compared with 757 μmol·h/L when receiving NaPBA. Side effects were mostly mild; none were regarded as related to NaPBA nor GPB. One patient on each of NaPBA and GPB experienced hyperammonaemia and withdrew from the study. Fourteen participants continued on GPB, and one additional participant was recruited. At the 12-month data cut-off point, 14 participants were receiving GPB. Ammonia levels during the extension remained within normal levels. Two episodes of side effects (nausea and QT prolongation) were considered treatment-related, whereas one severe event (gastroenteritis norovirus) and two episodes of hyperammonaemia were deemed unrelated to treatment. GPB provided a practical and clinically advantageous alternative to NaPBA, extending previous evidence to Japanese individuals with UCDs.

Who should read this article?

This summary may be useful for people living with urea cycle disorders, their families and caregivers. Patient advocates and healthcare professionals may also find this summary useful.

What are Urea Cycle Disorders?

The urea cycle is a metabolic pathway which primarily occurs in the liver to remove excess nitrogen from the body. The urea cycle removes nitrogen by converting toxic ammonia into harmless urea which can be transported to and filtered through the kidneys to eventually be excreted from the body via urine. Within the urea cycle, there are six enzymes and two transporters required to facilitate the breakdown of ammonia into urea. Urea Cycle Disorders (UCDs) are rare inherited genetic conditions caused by a deficiency in one of these enzymes or the transporters of the urea cycle.

The eight UCDs and their associated deficiencies are:

Disorder	Enzyme deficiency
**Arginase Deficiency**	Arginase (ARG)
**Carbamyl Phosphate Synthetase Deficiency**	Carbamoyl Phosphate Synthetase (CPS)
**Citrullinaemia**	Argininosuccinate Synthetase (ASS)
**Ornithine Transcarbamylase Deficiency**	Ornithine Transcarbamylase (OTC)
**Argininosuccinic Aciduria (ASA)**	Argininosuccinate Lyase (ASL)
**N-Acetylglutamate Synthetase Deficiency**	N-Acetylglutamate Synthetase (NAGS)

Disorder	Transporter deficiency
**Ornithine Transporter Deficiency**	Ornithine Translocase (ORNT1)
**Citrullinaemia Type II**	Citrin

UCDs can lead to very high levels of ammonia in the blood (hyperammonaemia) and other symptoms include vomiting, poor appetite, rapid breathing, seizures, altered consciousness, behavioural abnormalities, and developmental retardation. In severe cases, UCDs can be life-threatening. While many cases present in infancy, some individuals are diagnosed in adulthood.

Long-term treatment of UCDs includes dietary restriction of protein to minimise the accumulation of ammonia and other nitrogen waste in the body; this can cause many UCD patients to inherently avoid protein or self-limit their intake due to their fear of hyperammonaemia. As a result, many UCD patients fail to achieve the safe level of protein intake (SLPI) required for growth and preserving lean muscle mass. To this end, nitrogen-scavenging agents are used in the management of UCDs to buffer fluctuations in nitrogen load and help patients achieve their SLPI.

What treatments were studied?

Sodium phenylbutyrate (NaPBA) is a nitrogen-scavenging drug used for the long-term management of UCD. NaPBA binds to nitrogen and facilitates its excretion through urine. This is an alternative route to the urea cycle for excess nitrogen elimination. While NaPBA improves waste nitrogen excretion, it has several limitations: poor palatability, high dosing volume, and high sodium content.

Glycerol phenylbutyrate (GPB) is a **prodrug** of PBA that was developed to address the limitations of NaPBA. GPB is sodium-free, more concentrated volume-wise, and tasteless.

> 'Prodrugs' are medications that are pharmacologically inactive until they are metabolised, whereby they are converted into an active drug inside the body.

Why was the study done?

Clinical trials conducted in the US and Canada demonstrated that the efficacy of GPB was at least equivalent to that of NaPBA, and that its safety and tolerability were confirmed. However, to date, no clinical trials had examined GPB in Japanese patients with UCDs. Variations in genetic background may influence how GPB behaves within the body and how it affects clinical outcomes.

Who took part in the trial?

Seventeen participants were enrolled into an initial 14-day switch-over phase of the study. Two patients withdrew because of hyperammonaemia. Fifteen participants (14 from the switch-over, 1 newly recruited) were enrolled in an extension phase and 14 were still receiving treatment with GPB at 12 months. To take part in this study, patients had to:

- ✓ Be any age and sex with a confirmed diagnosis of UCD.
- ✓ Have been on a stable dose of NaPBA for at least one week prior to start of study.
- ✓ Not received sodium benzoate within one week prior to study drug administration.

Participants were excluded from the study if they:

- × Had very high blood ammonia levels at the start of the study.
- × Had recently experienced symptoms caused by high ammonia levels.
- × Had an active infection or other medical conditions that could affect how ammonia is processed in the body.
- × Had health problems or test results that may increase the risk of taking part.
- × Were using, or planned to use, medicines that could affect kidney function, protein breakdown, or blood ammonia levels.
- × Had a known heart rhythm condition.
- × Had previously had an allergic reaction to phenylbutyrate or related substances.
- × Had previously received a liver transplant.

Participant Characteristics

Switch-over Phase

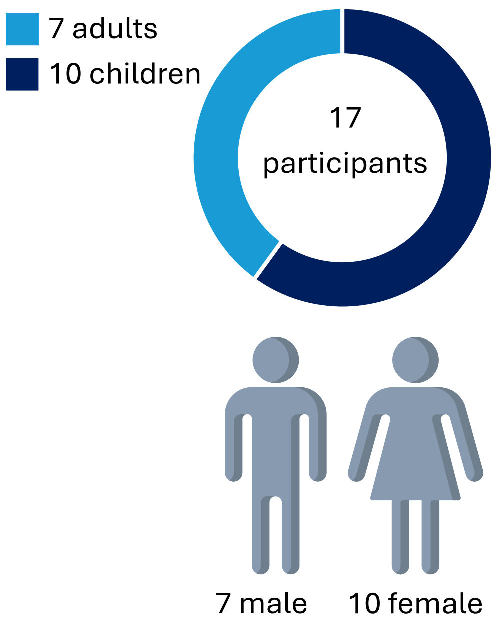

12-month Extension Phase

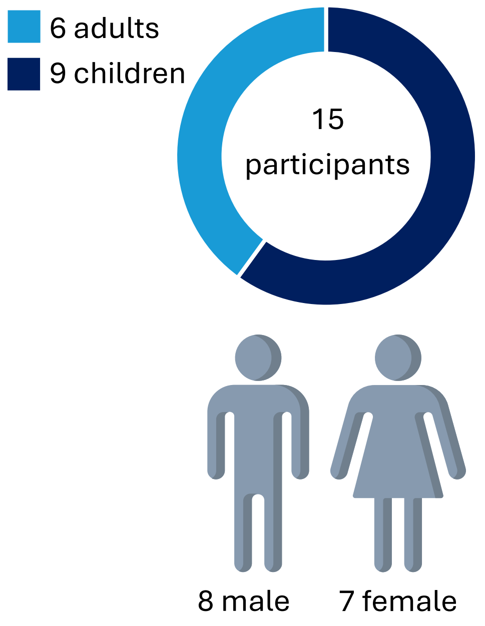

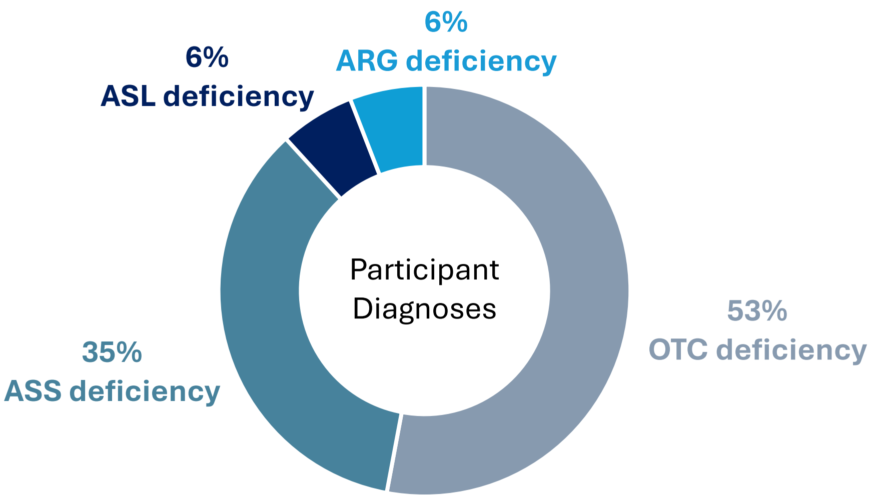

What did the study involve?

This was a phase 3, open-label, switch-over with extension study.

Phase 3

Phase 3 studies are conducted after successful completion of Phase 1 and Phase 2 studies.

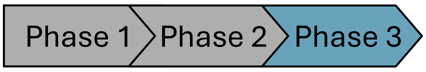

Phase 1 studies test the safety of the drug in healthy people. Phase 2 studies test efficacy and safety in a small group of people with the disorder. Phase 3 studies test how effective and well tolerated the drug is over longer periods of time

Open-label

Open-label is a type of study whereby both the participant and the healthcare professional are aware of the drug treatment being given.

Switch-over with extension phase

This was a switch-over study with an extension phase. The first phase of the study, the 'switch-over phase' involved 17 participants receiving NaPBA for 7 days then switching to GPB for days 8 to 14. Both drugs were given to participants orally three times a day, with or immediately after meals. Fifteen participants completed the switch-over. During the 'extension phase', 15 participants (1 newly enrolled, 14 from the switch-over phase) received GPB, with 14 participants remaining on treatment with GPB at Month 12.

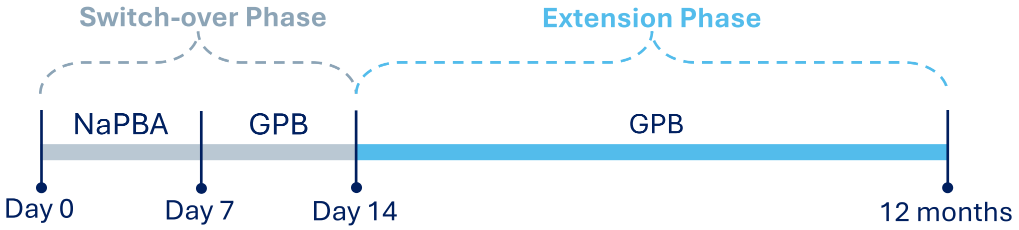

What did the study investigate?

UCD patients have a deficiency in an enzyme or transporter that breaks down ammonia, meaning they are prone to a build-up of ammonia exceeding normal levels.

During the switch-over phase, this study looked at the effectiveness of NaPBA and GPB by measuring blood ammonia concentration over 24 hours on Day 7 (end of NaPBA treatment), and on Day 14 (end of GPB treatment). Then to assess how well tolerated and effective GPB is long-term, blood ammonia concentrations were periodically assessed for 12-months during the extension phase.

Ammonia level data from the different laboratories involved in the study were normalised to a standard laboratory reference range before conducting the analyses, to allow for fair comparisons across study sites.

What were the main findings of the study?

The study began in April 2023 and was conducted at 9 clinical sites across Japan.

After switching to GPB, average ammonia exposure measured over 24 hours was 627 µmol·h/L compared with 757 µmol·h/L when receiving NaPBA.

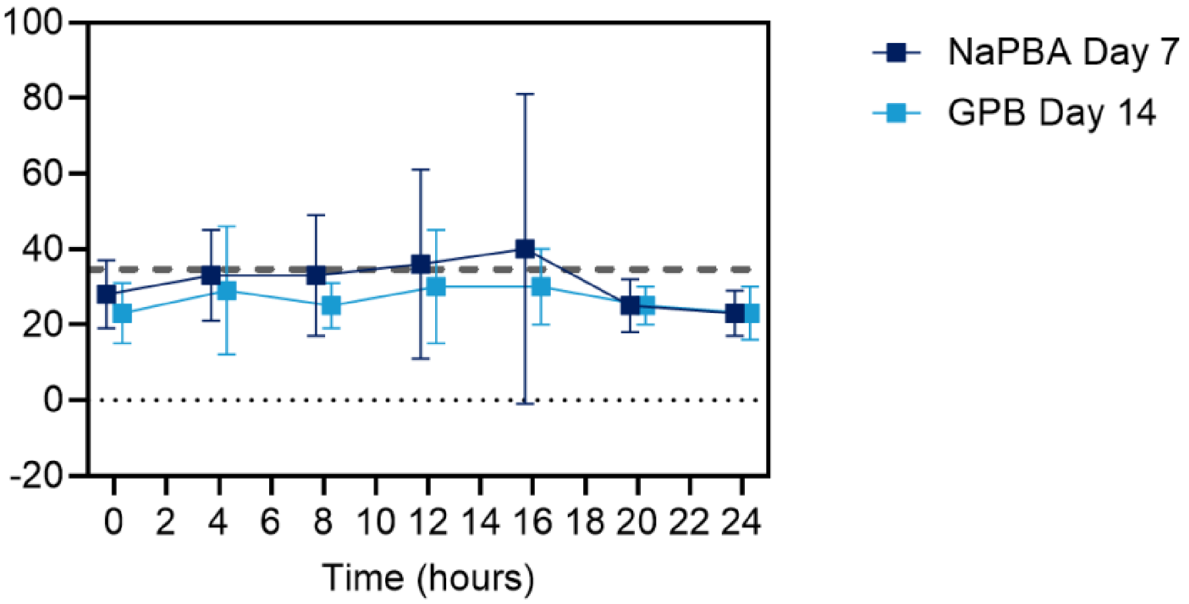

Figure 1. Average blood ammonia concentration across 24-hour period with dashed line indicating a standardised upper limit of normal of 35 µmol/L

During the 12-month extension phase, the average blood ammonia levels remained within the normal range (11 to 35 μmol/L), showing sustained control over time.

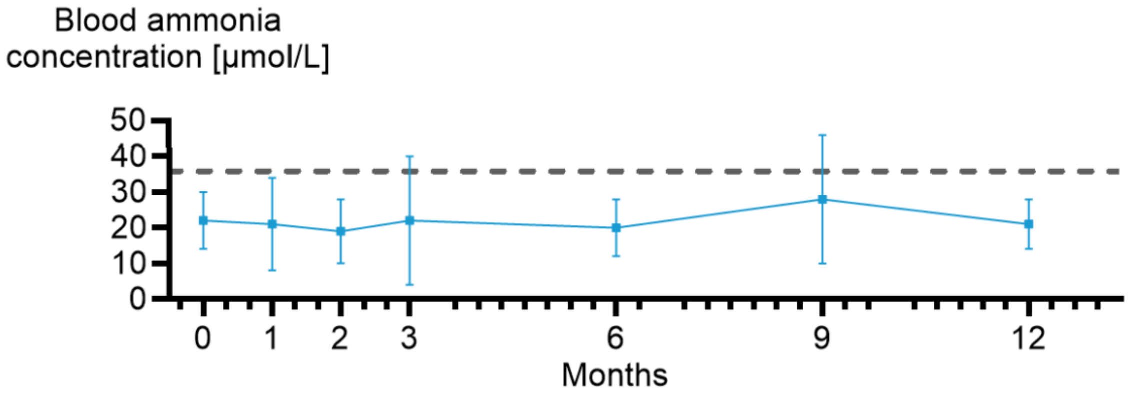

Figure 2. Average blood ammonia concentration through-out 12-month follow-up with dashed line indicating upper limit of normal (35 μmol/L).

Any safety concerns?

Side effects were mostly mild to moderate. Within the switch-over phase, one patient experienced hyperammonaemia during NaPBA treatment and another patient experienced hyperammonaemia during GPB treatment; both participants left the study. During the extension-phase, no patients discontinued the study due to adverse events and no deaths were reported. Two episodes of side effects (nausea and QT prolongation) were considered treatment-related, whereas one severe event (gastroenteritis norovirus) and two episodes of hyperammonaemia were deemed unrelated to treatment.

No clinically significant abnormalities were observed in laboratory parameters, ECGs, or amino acid profiles throughout the whole study.

What do the results of this study mean?

NaPBA remains the most widely used nitrogen scavenger treatment for UCDs in Japan. However, this study supports a transition to GPB where clinically indicated, showing that it can provide tight ammonia control without new safety concerns being identified. The switch-over phase of this study demonstrates that patients currently on stable NaPBA therapy can be transitioned to GPB.

The authors speculate that GPB's improved palatability, low dosing volume, and reduced sodium burden may contribute to improved patient adherence, thereby enabling satisfactory long-term control of ammonia levels.

Is this treatment available?

On December 22, 2025, GPB, known by the brand name Ravicti®, received marketing approval in Japan.

Limitations?

Due to the rarity of UCDs in Japan (1 in 50,000 births), this study investigated a small population size. The study was also open-label which means both the clinical staff and the participants knew which treatment they were taking.

Who sponsored the study?

This study was funded by OrphanPacific Inc. Some authors are employees of the sponsor. Trial registration: jRCT2071220110.
